# Propensity-Score-Matched Evaluation of Adverse Events Affecting Recovery after COVID-19 Vaccination: On Adenovirus and mRNA Vaccines

**DOI:** 10.3390/vaccines10020284

**Published:** 2022-02-13

**Authors:** Chang-Sik Son, Sang-Hyeon Jin, Won-Seok Kang

**Affiliations:** Division of Intelligent Robot, Daegu Gyeongbuk Institute of Science and Technology (DGIST), Daegu 42988, Korea; jinjinsh@dgist.ac.kr

**Keywords:** COVID-19 vaccine, adenovirus vector, mRNA, VAERS, adverse event, propensity score matching

## Abstract

This study aimed to observe adverse events following immunisation (AEFIs) that affected recovery within two weeks after COVID-19 vaccination and investigate their risks in propensity-score-matched populations. Data were collected from 447,346 reports from the VAERS between 1 January 2021 and 31 July 2021. Propensity-score-matched populations were constructed by adjusting for demographic characteristics and 11 underlying diseases in eligible subjects who received 1 of 3 COVID-19 vaccines: 19,462 Ad26.COV2.S, 120,580 mRNA-1273, and 100,752 BNT162b2. We observed that 88 suspected AEFIs (22 in Ad26.COV2.S, 62 in mRNA-1273, and 54 in BNT162b2) were associated with an increased risk of delayed recovery within 2 weeks after COVID-19 vaccinations. Nervous system, musculoskeletal and connective tissue, gastrointestinal, skin, and subcutaneous tissue disorders were the most common AEFIs after COVID-19 vaccination. Interestingly, four local and systemic reactions affected recovery in different vaccine recipients during our study period: asthenic conditions and febrile disorders in Ad26.COV2.S and mRNA-1273; general signs and symptoms in mRNA-1273 and BNT162b2; injection site reactions in Ad26.COV2.S and BNT162b2. Although it is necessary to confirm a causal relationship with COVID-19 vaccinations, some symptoms, including paralysis, allergic disorders, breathing abnormalities, and visual impairment, may hinder the recovery of these recipients.

## 1. Introduction

At the end of 2019, a novel coronavirus, which is now known as severe acute respiratory syndrome coronavirus 2 (SARS-CoV-2), was identified as the cause of a cluster of pneumonia cases in Wuhan, a city in the Hubei Province of China [1]. The rapidly expanding COVID-19 pandemic has impacted all areas of daily life. As of 3 June 2021, COVID-19 outbreaks were reported in 213 countries, with over 171 million confirmed cases and more than 3.68 million deaths [2].

Various vaccines to prevent SARS-CoV-2 infection are available in different countries. In the United States, COVID-19 mRNA vaccines—BNT162b2 (Pfizer-BioNTech COVID-19 vaccine), mRNA-1273 (Moderna COVID-19 vaccine), and COVID-19 adenovirus vector vaccine Ad26.COV2.S (Janssen COVID-19 vaccine)—received emergency use authorisation for use in individuals 18 years and older. BNT162b2 is also authorised for use in children and adolescents 12–17 years old [3,4,5]. In large placebo-controlled trials, these vaccines were shown to be highly effective in preventing laboratory-confirmed COVID-19, especially in patients with severe or critical disease [6,7,8].

After COVID-19 vaccination, local reactions, such as pain or redness at the injection site, were mild. Moderate systemic reactions, such as fatigue, myalgia, arthralgia, and headache, were observed in less than 50% of mRNA vaccine recipients, starting about 15 h after vaccination and resolving in most recipients by day 2 without sequelae [6,7]. However, very rare but severe vaccine-associated adverse events (AEs) have occurred, such as anaphylaxis [9] and myocarditis [10] with mRNA vaccines, and thrombocytopenia [11] and Guillain–Barre syndrome [12] with Ad26.COV2.S. Possible facial nerve paralysis [7,13,14], including Bell’s palsy, has been reported among mRNA vaccine recipients.

There are reports of unknown and unverified moderate-to-severe adverse reactions after COVID-19 vaccination, but a causal relationship has not been medically confirmed. Therefore, real-world monitoring of vaccine safety is crucial to help understand the suspected AEs following immunisation (AEFIs), assess their incidence, and determine risk factors to inform potential vaccine contraindications [15]. There are two monitoring systems in place to assess safety in post-licensure settings: the Vaccine Adverse Event Reporting System (VAERS), which is a passive system that relies on others reporting adverse events; and the Vaccine Safety Datalink, which is an active system that reviews databases or conducts studies to identify AEs [16]. The VAERS is a vaccine surveillance programme that is co-managed by the US Centre for Disease Control and Prevention and the US Food and Drug Administration, which is responsible for monitoring the safety of pharmaceutical drugs after being released to the market. Collecting useful information on AEs from various vaccines provides many advantages [17,18,19]: (1) detecting new, unusual, or rare AEs; (2) monitoring reporting trends that might reflect true increases in known AEs; (3) identifying potential risk factors for particular AEs; and (4) detecting persistent use safety problems and administration errors.

Recently, several studies investigated moderate-to-severe [15] or serious AEs (e.g., death and hospitalisation) [20] and underlying health conditions associated with deaths [15,21] among COVID-19-vaccinated cases. For example, the deaths reported by the VAERS were in vaccinated people aged 85 and older at long-term care facilities. These patients were frail older people with serious underlying diseases, including hypertension, diabetes, anaemia, heart failure, and chronic obstructive pulmonary disease [21]. Furthermore, other underlying diseases, such as obesity (i.e., body mass index > 30 kg/m^2^) [22,23,24,25], chronic kidney disease [25,26,27,28], cancer [25,29], and allergy [9,30,31], are known possible epidemiologic risk factors for severe COVID-19.

To examine various COVID-19-vaccine-induced AEs, we collected comprehensive data including 1122 AEFIs from three COVID-19 vaccines (i.e., Ad26.COV2.S, mRNA-1273, and BNT162b2), reported to the VAERS from 1 January 2021 to 31 July 2021. AEs were examined in different propensity-score-matched populations, adjusted for demographic characteristics (e.g., age and sex) and 11 underlying diseases (e.g., obesity, hypertension, diabetes, heart failure, pulmonary disease, stroke, allergy). Major AEs and their adjusted hazard ratios were estimated using a multivariate Cox regression model.

## 2. Materials and Methods

### 2.1. Data Source and Collection

We used a dataset of vaccinated cases from the USA containing various types of vaccine-related information. The raw dataset comprised three different files collected by the VAERS database [17,18,19] for subjects vaccinated from 1 January 2021 to 31 July 2021. The VAERS data included demographic and medical history, symptoms for vaccine-associated AEs, and type of vaccine used. In the raw dataset, we collected all cases classified with the COVID-19 vaccine type. There were some cases with the same identification number (ID), and the four vaccine manufacturers were Janssen, Moderna, Pfizer-BioNTech, and Unknown. We considered medical records including general information (i.e., VAERS ID, age, sex, current illness, and medical history at the time of vaccination), vaccination information (i.e., vaccine type, manufacturer, vaccination date, and AE onset date), and recovery from vaccine-induced AEs.

**Figure 1:**
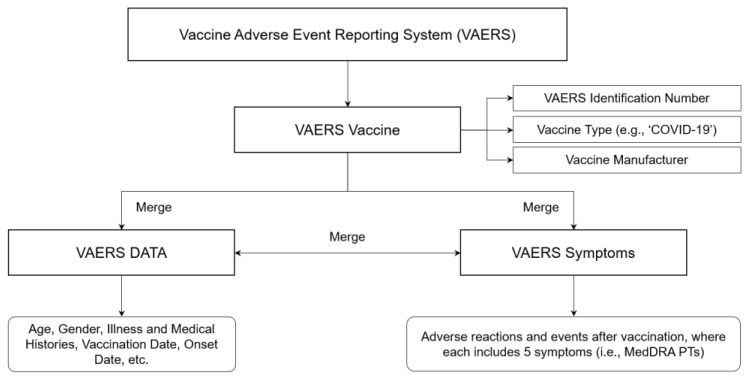
Data collection process.

As illustrated in Figure 1, the three files used a common VAERS ID as the primary key. To extract all cases of COVID-19 vaccination, the VAERS IDs were initially screened and used to extract some medical information, such as demographics, prediagnosed illness or medical histories, and AEs for individuals who were vaccinated against COVID-19. In particular, AEs were defined using standard medical terms in *The Medical Dictionary for Regulatory Activities* (MedDRA) [32]. Each case had a maximum of five terms, with each term listed for the corresponding VAERS ID.

### 2.2. Standard Medical Terms for Vaccine Adverse Events

MedDRA is organised in a five-level hierarchy. The highest or broadest level is the system organ class (SOC), which is further divided into high-level group terms (HLGT), high-level terms (HLT), preferred terms (PT), and the most granular lowest-level terms (LLT); the hierarchy includes 27, 337, 1737, 24,820, and 83,291 terms in release version 24.0 (March 2021) [33], respectively.

In cases reported to the VAERS database, various AEFIs were provided at the PT level. However, PTs are highly fragmented into signs, symptoms, diagnosis, investigation, or medical procedures, which might lead to failures in identifying differences in the AE incidence [34,35]. Accordingly, we converted the standard terms associated with all AEFIs (expressed as PTs) into HLTs. We collected 1122 COVID-19-vaccine-induced AEs and analysed their relationships at the SOC level. Each AE encoded a binary value indicating whether the COVID-19 vaccine recipient had certain adverse symptoms, where 1 indicated YES and 0 indicated NO.

### 2.3. Underlying Diseases

We examined medical records related to individual medical histories (i.e., prediagnosed illnesses) at the time of COVID-19 vaccination. Among these records, we extracted 11 underlying disabilities associated with risk factors for severe COVID-19 by using the keyword matching approach that considers representative terms for each illness. The underlying diseases were obesity [22,23,24,25], hypertension [25,36,37], diabetes [25,37], atrial fibrillation [25,38], heart failure [25,39,40], kidney disease [25,26,27,28], pulmonary disease [25,37,41], stroke [42], asthma [25,43,44], cancer [25,29], and allergies [9,30,31]. These terms were selected based on a literature review of previous studies.

### 2.4. Study Design

**Figure 2:**
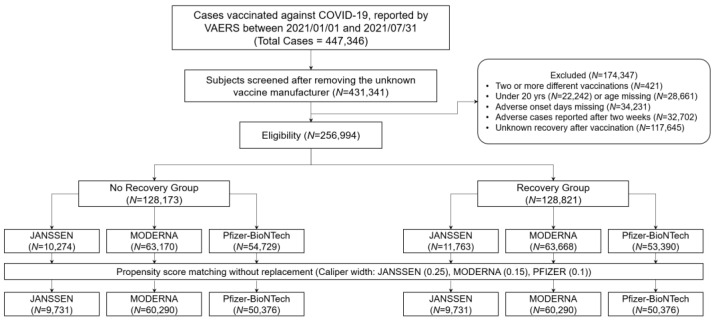
Study workflow.

The study population included 447,346 cases: 45,848 for Janssen vaccination, 197,006 for Moderna vaccination, 203,478 for Pfizer-BioNTech vaccination, and 1014 for unknown. After excluding unknown COVID-19 vaccinations, 431,341 individuals with a unique VAERS ID were screened. The primary endpoint was all adverse symptoms reported within two weeks after COVID-19 vaccination. Accordingly, we considered 256,994 individuals as eligible subjects according to the following exclusion criteria: 2 or more different COVID-19 vaccinations; missing age data or under 20 years of age; missing data on the number of days associated with vaccine-related adverse events; cases reported after two weeks; or unreported recovery from vaccine-induced AEs. Study subjects were categorised into 7 and 3 groups according to age and gender, respectively: (1) 20–29, 30–39, 40–49, 50–59, 60–69, 70–79, and ≥80 years; (2) unknown, male, and female. After propensity score matching, 19,462, 120,580, and 100,752 individuals were included in the analysis, as illustrated in Figure 2. This workflow shows the numbers of individuals (N) excluded at different stages and the number of cases before/after propensity score matching.

### 2.5. Statistical Analysis

#### 2.5.1. Propensity Score Matching

Data pertaining to our study population collected from the VAERS database includes various age groups, genders, and various underlying diseases for each individual at the time of COVID-19 vaccination. The type of vaccine used might depend on individual characteristics, including age, sex, and comorbidities, and this choice could affect the vaccine-associated AEs and recovery from them. Therefore, we used propensity score matching (PSM) using the nearest neighbour method with a 1:1 matching ratio without replacement, to reduce the bias due to these confounding variables between two groups (i.e., recovery and no recovery from COVID-19-vaccine-induced AEs). The propensity score, specifically the conditional probability of not recovering from COVID-19-vaccine-induced AEs, given the observed covariates (i.e., in our study—age, sex, and 11 underlying diseases), was estimated through binomial logistic regression analysis. To rigorously control for confounding variables, the calliper width (i.e., considered only if the difference in the propensity score between paired subjects was within a prescribed range) was used during the PSM. The calliper threshold was examined between 0.25 and 0.1 of the standard deviation of the propensity score. A chi-square test was used as a criterion to determine the best calliper threshold, at which all covariates had no statistically significant difference (*p* > 0.05). The absolute standardised difference (ASD) [45] was set below 0.1 to confirm whether the two groups were well balanced.

#### 2.5.2. Cox Proportional Hazards Regression Analysis

We considered 1122 COVID-19-vaccine-associated AEs (i.e., 753, 1019, and 1000 MedDRA HLTs for Ad26.COV2.S, mRNA-1273, and BNT 162b2, respectively) as independent variables, reported within 2 weeks from individuals being vaccinated with 1 type of the 3 vaccines. We examined the association between those AEs and recovery using a Cox proportional hazards regression analysis. The event of interest was defined as individuals who did not recover from the vaccine-induced AEs during our study period. We removed some AEs with a small sample size to resolve any monotonic likelihood issues that could occur in Cox proportional hazards regression analysis. These issues are observed when fitting a Cox model when at least one covariate estimate diverges to negative or positive infinity [46]. Accordingly, we selected AEs including the minimum number of cases with more than five adverse symptoms within the event (i.e., no recovery group from vaccine-induced AEs). Based on this criterion, we performed univariate Cox proportional hazards regression analysis to screen for possible AEs that delayed recovery after COVID-19 vaccination. Statistically significant AEs were included in the multivariate Cox regression model for adjustment. The results of the Cox regression model are presented as HRs with 95% confidence intervals. Statistical significance was set at *p* < 0.05. The concordance index or C-index was used to identify the discrimination power of the multivariate Cox regression model.

#### 2.5.3. Experimental Environment and Implementation

All experiments were implemented and evaluated using the following hardware and software: AMD Ryzen 9 3900X 12-Core Processor @ 4.19GHz CPU; 128GB RAM; Windows 10; IntelliJ IDEA 2019.2.4 (Ultimate Edition) using Python (version 3.7); numpy (version 1.19.1), pandas (version 1.0.3), scikit-learn (version 0.22.2), scipy (version 1.4.1), statsmodels (version 0.12.2), and lifeline (version 0.26.0).

## 3. Results

### 3.1. Data Characteristics

Among the 256,994 individuals, the average age of each group was 50.01 ± 16.59 years: 44.69 ± 15.1 for Ad26.COV2.S; 51.45 ± 16.76 for mRNA-1273; and 49.4 ± 16.43 for BNT162b2. The total gender distribution was 718 unknown (0.28%), 66,280 men (25.79%), and 189,996 women (73.93%). The proportion of women was higher than that of men (approximately 66% to 76%). Three underlying disabilities, namely hypertension (8.31%), asthma (4.87%), and diabetes (4.66%), were the most common medical history terms or prediagnosed illnesses of individuals at the time of vaccination. In particular, the proportion was higher in the mRNA-1273 vaccine than the other two vaccines. Moreover, the AE onset interval for each COVID-19 vaccine was 2.07 ± 3.18 days: 1.67 ± 3.1 for Ad26.COV2.S; 2.51 ± 3.43 for mRNA-1273; and 1.64 ± 2.8 for BNT162b2. The AE onset interval of mRNA-1273 vaccine recipients was approximately 0.84–0.87 days longer than those of the other vaccine recipients. The results revealed that 128,821 (50.13%) recovered and the remaining 128,173 (49.87%) did not recover from AEFIs (Table 1).

**Figure 3:**
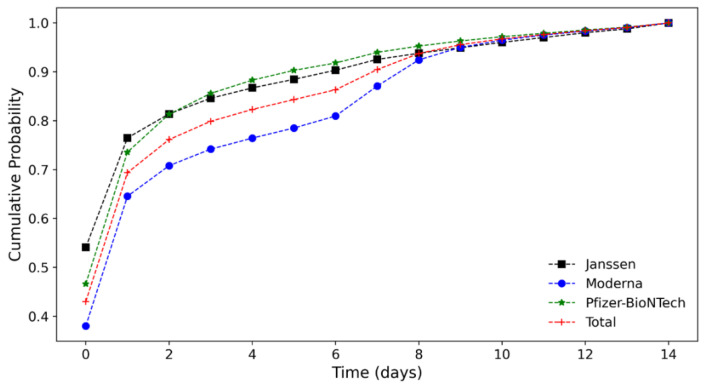
Cumulative probability of AE onset following COVID-19 vaccination.

Figure 3 shows the cumulative probability of AE onset for two weeks after vaccination with the three COVID-19 vaccines. Approximately 70% of the patients experienced local and systemic adverse reactions to the vaccine within one day, such as injection site pain, headache, muscle-related symptoms, fever, nausea, or vomiting. For Ad26.COV2.S, mRNA-1273, and BNT162b2, AE onset was observed in 11,918 (54.08%), 48,174 (37.98%), and 50,392 (46.61%) individuals on day 0 and 4936 (22.4%), 33,685 (26.56%), and 29,146 (26.96%) individuals on day 1, respectively. As noted in previous studies, there was a decreasing trend thereafter [6,7,8].

### 3.2. Major AEs and the Risk Levels Associated with the Three COVID-19 Vaccines

#### 3.2.1. Janssen COVID-19 Vaccine

The distribution of demographic characteristics and the 11 underlying disabilities between the non-recovered and recovered groups, before and after PSM, are shown in Table S1. Before PSM, there were significant differences (*p* < 0.05) in age and sex. A total of 9 underlying diseases—namely hypertension (7.92%), asthma (5.63%), diabetes (4.55%), allergies (2.88%), obesity (1.54%), pulmonary disease (1.31%), cancers (1.08%), heart failure (0.8%), and kidney disease (0.45%)—were relatively high in the non-recovered group, with statistically significant differences compared with the recovered group (*p* < 0.05). After PSM, 2 groups were paired with a 1:1 matching ratio and the calliper width was adjusted to 0.25. This caused the differences between these groups to disappear (ASD < 0.1, *p* > 0.05).

In the propensity-score-matched population, 91 adverse symptoms were associated with the risk of developing an AE estimated with a univariate Cox proportional hazard regression model. The concordance index of a multivariate Cox regression model was then 0.6518. Overall, 36 AEs were associated with the Janssen COVID-19 vaccination (see Figure S1). Of these, 22 were major vaccine-induced AEs with extended recovery times. The adjusted HRs were distributed from 1.06 to 2.6 and grouped into 11 SOC terms (Table 2).

Among the SOCs, five were common AEFIs, such as nervous system disorders (38.73%), general disorders and administration site conditions (29.05%), musculoskeletal and connective tissue disorders (23.71%), gastrointestinal disorders (20.18%), and skin and subcutaneous tissue disorders (15.26%). In particular, some AEs—headaches (36.23%; HR, 1.16; 95% CI, 1.11–1.22), asthenic conditions (29.61%; HR, 1.06; 95% CI, 1.01–1.12), pain and discomfort (26.1%; HR, 1.17; 95% CI, 1.12–1.23), febrile disorders (25.94%; HR, 1.2; 95% CI, 1.14–1.27), musculoskeletal and connective tissue pain and discomfort (19.87%; HR, 1.12; 95% CI, 1.06–1.18), nausea and vomiting symptoms (19.09%; HR, 1.11; 95% CI, 1.05–1.18), and dermal and epidermal conditions (12.99%; HR, 1.08; 95% CI, 1.02–1.15)—generally exhibited an increased risk of non-recovery in individuals compared with recovered people who received the Ad26.COV2.S vaccine. The remaining six SOCs were also rare AEFIs. Some of these, such as allergic conditions (2.13%; HR, 1.27; 95% CI, 1.1–1.46), cognitive and attention disorders and disturbances (1.2%; HR, 1.21; 95% CI, 1.01–1.46), pharyngeal disorders (0.69%; HR, 1.34; 95% CI, 1.05–1.72), and cholecystitis and cholelithiasis (0.07%; HR, 2.6; 95% CI, 1.21–5.63), were identified as risk factors related to delayed recovery of vaccine recipients.

#### 3.2.2. Moderna COVID-19 Vaccine

Table S2 shows the distribution of demographic characteristics and 11 underlying disabilities between individuals vaccinated with the Moderna vaccine in the non-recovered and recovered groups before and after PSM. Before PSM, there were significant differences (*p* < 0.05) in age and sex. Nine underlying diseases, namely, hypertension (10.16%), diabetes (5.79%), asthma (5.38%), allergies (2.81%), obesity (1.77%), cancers (1.47%), pulmonary disease (1.23%), heart failure (0.95%), and stroke (0.25%), were relatively high in the non-recovered group, with statistically significant differences compared with the recovered group (*p* < 0.05). After PSM, two groups were paired with a 1:1 matching ratio, the calliper width was adjusted to 0.15, and the differences between these groups disappeared (ASD < 0.1, *p* > 0.05).

In the propensity-score-matched population, we identified 193 adverse symptoms associated with the risk of developing an AE, estimated with a univariate Cox proportional hazard regression model. The concordance index of a multivariate Cox regression model was 0.6358. Overall, 94 AEs were associated with the Moderna COVID-19 vaccine (see Figure S2). Of these, 62 were major vaccine-induced AEs; their HRs ranged from 1.05 to 3.47 and were grouped into 21 SOC terms (Table 3).

A total of 6 AEFIs—general disorders and administration site conditions (27.29%), nervous system disorders (24.24%), musculoskeletal and connective tissue disorders (19.11%), gastrointestinal disorders (13.75%), skin and subcutaneous tissue disorders (12.09%), and cardiac disorders (10.98%)—were the most common (similar to the Janssen vaccine results). However, the detailed AEs and their distribution were different in the non-recovered group. Among these, some AEs exhibited increased risk of non-recovery cases: (1) asthenic conditions (20.65%; HR, 1.06; 95% CI, 1.03–1.08), pain and discomfort (19.28%; HR, 1.16; 95% CI, 1.02–1.35), febrile disorders (15.86%; HR, 1.14; 95% CI, 1.11–1.17), and general signs and symptoms (15.7%; HR, 1.1; 95% CI, 1.07–1.12); (2) headaches (19.41%; HR, 1.2; 95% CI, 1.17–1.23); (3) musculoskeletal and connective tissue pain and discomfort (16.09%; HR, 1.16; 95% CI, 1.13–1.18), joint-related signs and symptoms (8.65%; HR, 1.25; 95% CI, 1.21–1.29), and muscle pain (6.17%; HR, 1.05; 95% CI, 1.02–1.09); (4) nausea and vomiting symptoms (11.71%; HR, 1.07; 95% CI, 1.04–1.1); (5) dermal and epidermal conditions (12.09%; HR, 1.07; 95% CI, 1.05–1.1); (6) cardiac signs and symptoms (9.05%; HR, 1.07; 95% CI, 1.04–1.1). We also identified rare AEs with relatively increased risk in the non-recovered group when compared with the recovered group. In particular, decreased fluid intake (0.03%, HR, 1.83; 95% CI, 1.14–2.94), hypoglycaemic conditions (0.02%; HR, 2.13; 95% CI, 1.21–3.73), hepatobiliary signs and symptoms (0.02%; HR, 1.86; 95% CI, 1.11–3.11), clostridia infections (0.02%; HR, 3.47; 95% CI, 2.02–5.94), and chest and respiratory tract injuries (0.01%; HR, 2.42; 95% CI, 1.21–4.85) were observed.

#### 3.2.3. Pfizer-BioNTech COVID-19 Vaccine

Table S3 shows the demographic characteristics and 11 underlying disabilities between persons treated with the Pfizer vaccine in the non-recovered and recovery groups before and after PSM. Before PSM, there were significant differences (*p* < 0.05) in age and sex. A total of 3 underlying diseases—diabetes (4.24%), pulmonary disease (0.86%), and heart failure (0.7%)—were relatively higher in the non-recovered group than in the recovered group (*p* < 0.05). However, asthma (4.26%) exhibited a relatively low distribution, with a statistically significant difference (*p* < 0.05) in the non-recovered compared with the recovered group. After PSM, 2 groups were paired using a 1:1 matching ratio and the calliper width was adjusted to 0.1. This caused the differences to disappear between these groups (ASD < 0.1, *p* > 0.05).

In the propensity-score-matched population, we observed 208 adverse symptoms associated with the risk of developing an AE estimated with a univariate Cox proportional hazard regression model. The concordance index of a multivariate Cox regression model was 0.6237. Overall, 92 AEs were associated with Pfizer-BioNTech COVID-19 vaccination (see Figure S3). Of these, 54 were the major vaccine-induced AEs; their hazard ratios ranged from 1.04 to 3.37 and were grouped into 16 SOC terms (Table 4).

A total of 5 adverse reactions were more common in the non-recovered group: general disorders and administration site conditions (31.8%), nervous system disorders (30.13%), musculoskeletal and connective tissue disorders (19.55%), gastrointestinal disorders (17.97%), and skin and subcutaneous tissue disorders (15.05%). Among these, 7 AEs—including headaches (22.9%, HR, 1.13; 95% CI, 1.11–1.16), pain and discomfort (19.47%; HR, 1.08; 95% CI, 1.05–1.11), musculoskeletal and connective tissue pain and discomfort (15.98%; HR, 1.19; 95% CI, 1.16–1.22), nausea and vomiting symptoms (13.76%; HR, 1.04; 95% CI, 1.01–1.07), general signs and symptoms (13.32%; HR, 1.07; 95% CI, 1.04–1.1), dermal and epidermal conditions (11.3%; HR, 1.2; 95% CI, 1.16–1.23), and joint-related signs and symptoms (10.22%; HR, 1.12; 95% CI, 1.09–1.16)—generally exhibited an increased risk of non-recovery compared with that in recovered people who received the BNT162b2 vaccine. Some other AEs—including lymphatic system disorders (7.41%; HR, 1.25; 95% CI, 1.21–1.29), breathing abnormalities (6.15%; HR, 1.12; 95% CI, 1.08–1.17), ischaemic coronary artery disorders (5.45%; HR, 1.05; 95% CI, 1.01–1.1), choroid and vitreous haemorrhages and vascular disorders (0.01%; HR, 3.37; 95% CI, 1.48–7.7), and cardiac infections (0.01%; HR, 2.72; 95% CI, 1.2–6.14)—were rare, but had elevated risk of non-recovery compared with that in recovered people who received the BNT162b2 vaccine.

## 4. Discussion

Our study focused on analysing major AEFIs and whether their risks affect the recovery after COVID-19 vaccination. We investigated all possible AEs of three COVID-19 vaccines based on data reported to the VAERS database from 1 January 2021 to 31 July 2021 and analysed their relationships for different SOCs.

Our findings showed that among the 21 SOCs, 5 terms (general disorders and administration site conditions, nervous system, musculoskeletal and connective tissue, gastrointestinal, and skin and subcutaneous tissue disorders) were associated with generally more common AEFIs in the propensity-score-matched populations. We confirmed that the most prevalent AEFIs were pain (Ad26.COV2.S, 26.1%; mRNA-1273, 19.28%; BNT162b2, 19.47%), headaches (Ad26.COV2.S, 36.23%; mRNA-1273, 19.41%; BNT162b2, 22.9%), joint-related signs and symptoms (Ad26.COV2.S, 10.5%; mRNA-1273, 8.65%; BNT162b2, 10.22%), muscle pain (Ad26.COV2.S, 10.34%; mRNA-1273, 6.17%; BNT162b2, 7.59%), musculoskeletal and connective tissue pain and discomfort (Ad26.COV2.S, 19.87%; mRNA-1273, 16.09%; BNT162b2, 15.98%), nausea and vomiting symptoms (Ad26.COV2.S, 19.09%; mRNA-1273, 11.71%; BNT162b2, 13.76%), and dermal and epidermal conditions (Ad26.COV2.S, 12.99%; mRNA-1273, 12.09%; BNT162b2, 11.3%). These conditions were observed in more than approximately 6% of the non-recovered cases who received each of the COVID-19 vaccines. A total of 4 adverse symptoms—namely, asthenic conditions (Ad26.COV2.S, 29.61%; mRNA-1273, 20.65%), general signs and symptoms (mRNA-1273, 15.7%; BNT162b2, 13.32%), febrile disorders (Ad26.COV2.S, 25.94%; mRNA-1273, 15.86%), and injection site reactions (Ad26.COV2.S, 11.2%; BNT162b2, 8.96%)—were regarded as the additional local and systemic reactions. Simultaneously, these were risk factors associated with recovery after COVID-19 vaccinations. These findings are consistent with the previous results demonstrating that local and systemic reactions were common [6,7,15]. Most were mild reactions that did not prevent daily activities and mainly occurred within the first two days after vaccination [47,48].

Rare neurological findings, especially facial paralysis, were noted in several previous studies [7,13,14,15]. A total of 7 cases suspected of facial paralysis were reported among 36,930 recipients during clinical phase 3 trials for mRNA vaccines [6,7]. A recent study demonstrated that neurological AEs after COVID-19 vaccination may be more than chance events, while reporting 18 cases of facial paralysis and other neurological events, such as syncope (37 cases) and seizure (12 cases), to the VAERS database in December 2020 [15]. Similar to the previous study, we identified that 200 recipients with suspected paralysis and paresis (excluding cranial nerve) did not recover after mRNA-1273 vaccination, even though they did not develop facial paralysis or Bell’s palsy. However, they showed a 1.17-fold increased risk compared with the recovered group. Seizure-related symptoms were observed in 169 and 222 non-recovered cases among recipients who received mRNA-1273 or BNT162b2, respectively; however, their risk degree was lower than that in the recovered group (See Figures S2 and S3).

Severe allergic reactions, including anaphylaxis, were very rarely reported as a crucial issue [30,31]. Anaphylaxis is diagnosed primarily based on clinical symptoms, signs, and a detailed description of the acute episode, including antecedent activities and events occurring within the preceding minutes–hours [49]. We did not find adverse cases and the risk of anaphylactic and anaphylactoid responses in the propensity-score-matched populations associated with mRNA vaccination. However, allergic conditions related to immune system disorders, including hypersensitivity reactions (type I, III, or IV), were adverse symptoms associated with adenovirus vector and mRNA vaccines. We identified that during our study period, 207, 751, and 716 cases were non-recovered after Ad26.COV2.S, mRNA-1273, and BNT162b2 vaccinations, respectively. Other adverse symptoms, such as angio-oedemas, yielded 1364 (2.26%) non-recovered cases after mRNA-1273 vaccination.

Another very rare but severe vaccine-associated AE involves unusual types of thrombotic events associated with thrombocytopenia. Thrombosis occurs at unusual sites, including cerebral venous sinuses and mesenteric vessels, and often at more than one site [11,50]. The pathogenetic mechanism underlying vaccine-induced immune thrombotic events was identified by Greinacher et al. [51]. The clinical features included increased D-dimer levels, decreased or normal fibrinogen levels, and the presence of antibodies against platelet factor 4 (PF4). High levels of antibodies against PF4 have been detected in the serum of almost all patients who received ChAdOx1 (AstraZeneca) vaccination [52]. Anti-PF4 antibodies, which mimic heparin-induced thrombocytopenia-like disease, cause platelet activation and may induce thrombosis in rare cases. Therefore, this pathophysiologic mechanism implies potential neurosurgical consequences that could be derived from at least the sequelae of cerebral sinus and vein thrombosis [51]. Most of these AEs occurred within 2 weeks after patients received the initial vaccine dose, especially in females under 60 years of age. In the US, the risk of this syndrome following Ad26.COV2.S vaccination was assessed as 3 cases per million overall, and 8.8 cases per million for women 30–49 years old [12]. Nevertheless, COVID-19 vaccine recipients should be made aware of the possible association and seek immediate care for signs and symptoms suggestive of thrombocytopenia or thrombotic complications, such as shortness of breath, chest pain, lower extremity oedema, persistent abdominal pain, unabatingly severe headache, blurred vision, and severe backache [53]. We observed some cases of peripheral embolism and thrombosis (143 in Ad26.COV2.S; 196 in mRNA-1273; 246 in BNT162b2) among recipients who received 1 of the 3 COVID-19 vaccines (See Figures S1–S3). Simultaneously, some AEFIs, such as pulmonary embolism and thrombosis (232 in mRNA-1273; 247 in BNT162b2) and thrombocytopaenia (35 in BNT162b2), were observed in non-recovered cases after mRNA vaccinations. However, their risk was lower in the recovery group after mRNA vaccination (See Figures S2 and S3). Furthermore, we confirmed that some AEFIs, such as breathing abnormalities (mRNA-1273, 4.3%; BNT162b2, 6.15%), visual impairment (mRNA-1273, 0.47%), and neurological visual problems (mRNA-1273, 1.2%; BNT162b2, 1.82%), might hinder the recovery of mRNA vaccine recipients. Some other symptoms were rare, but risk factors interfering with the recovery of vaccine recipients were: (1) ischaemic coronary artery disorders (Ad26.COV2.S, 6.72%; mRNA-1273, 3.34%; BNT162b2, 5.45%); (2) ocular disorders (Ad26.COV2.S, 1.98%; mRNA-1273, 1.08%; BNT162b2, 1.77%); (3) gastrointestinal and abdominal pain (Ad26.COV2.S, 5.26%; mRNA-1273, 1.87%; BNT162b2, 2.99%); (4) cognitive and attention disorders (Ad26.COV2.S, 1.2%; mRNA-1273, 0.69%; BNT162b2, 0.95%); and (5) disturbances in initiating and maintaining sleep (Ad26.COV2.S, 2.13%; mRNA-1273, 1.05%; BNT162b2, 1.57%).

Our study has several limitations. First, unlike clinical trials that report adverse reactions using standardised data collection procedures, the VAERS database includes various adverse cases [15] that may include reporting bias, over-reporting, or under-reporting of AEs [17], because clinicians are encouraged to report all AEFIs. Therefore, the actual mild–moderate or severe AEs may be rarer than our findings. Second, our study data may include individuals who received the SARS-CoV-2 test before our analytic period and exhibited some symptoms of SARS-CoV-2 infection. To reduce such potential bias, we attempted to demonstrate possible major AEFIs concerning each COVID-19 vaccine in the propensity-score-matched populations adjusted for age, sex, and 11 underlying diseases. However, unmeasured and residual confounding may have biased our estimates. For example, we did not know the individual occupation characteristics (e.g., virus exposure degree and protective equipment use) [54], race, and residence. Consequently, our study findings may involve unknown or unverified AEs, since the VAERS database does not provide medically confirmed or valid diagnostic evidence. Third, to investigate AEFIs related to COVID-19 vaccines, we used only limited covariates that satisfied a user-defined sample threshold in propensity-score-matched target outcomes. Even though it is possible to reduce the monotonic likelihood issue (i.e., the bias of maximum likelihood estimates) in the Cox regression model, further investigations will be necessary.

## 5. Conclusions

We found that an increased risk of local and systemic reactions (e.g., headache, joint-related symptoms, muscle pain, musculoskeletal and connective tissue pain, nausea or vomiting symptoms, dermal and epidermal conditions, and febrile disorders) was associated with delayed recovery in non-recovered cases of different propensity-score-matched populations when compared with the group that recovered after COVID-19 vaccination. Furthermore, we observed that some notable AEs, such as paralysis (excluding cranial nerve), allergic disorder, breathing abnormality, and visual impairment, might hinder the recovery of non-recovery cases; although, it is necessary to confirm a causal relationship with COVID-19 vaccination. In the future, we aim to perform an observational study on the effectiveness and side effects of heterologous prime–boost, particularly in preventing severe disease and infection with Delta (B.1.617.2) and Omicron (B.1.1.529) variants.

## Figures and Tables

**Table 1:** Demographic and clinical characteristics of all individuals vaccinated against COVID-19.

Variable	Ad26.COV2.S	mRNA-1273	BNT162b2	Total
Total, N (%)	22,037 (8.57)	126,838 (49.35)	108,119 (42.07)	256,994
Age, mean (SD)	44.69 ± 15.1	51.45 ± 16.76	49.4 ± 16.43	50.01 ± 16.59
Gender				
Unknown, N (%)	34 (0.15)	225 (0.18)	459 (0.42)	718 (0.28)
Male, N (%)	7438 (33.75)	30,161 (23.78)	28,681 (26.53)	66,280 (25.79)
Female, N (%)	14,565 (66.09)	96,452 (76.04)	78,979 (73.05)	189,996 (73.93)
Underlying disability				
Obesity, N (%)	280 (1.27)	2027 (1.6)	1378 (1.27)	3685 (1.43)
Hypertension, N (%)	1437 (6.52)	12,148 (9.58)	7760 (7.18)	21,345 (8.31)
Diabetes, N (%)	792 (3.59)	6899 (5.44)	4288 (3.97)	11,979 (4.66)
Atrial fibrillation, N (%)	34 (0.15)	364 (0.29)	287 (0.27)	685 (0.27)
Heart failure, N (%)	126 (0.57)	1073 (0.85)	676 (0.63)	1875 (0.73)
Kidney disease, N (%)	78 (0.35)	782 (0.62)	524 (0.48)	1384 (0.54)
Pulmonary disease, N (%)	187 (0.85)	1396 (1.1)	798 (0.74)	2381 (0.93)
Asthma, N (%)	1061 (4.81)	6580 (5.19)	4880 (4.51)	12,521 (4.87)
Stroke, N (%)	36 (0.16)	285 (0.22)	234 (0.22)	555 (0.22)
Cancers, N (%)	200 (0.91)	1768 (1.39)	1221 (1.13)	3189 (1.24)
Allergies, N (%)	559 (2.54)	3256 (2.57)	2557 (2.36)	6372 (2.48)
Recovery				
Yes, N (%)	11,763 (53.38)	63,668 (50.2)	53,390 (49.38)	128,821 (50.13)
No, N (%)	10,274 (46.62)	63,170 (49.8)	54,729 (50.62)	128,173 (49.87)
AE onset interval (days), mean (SD *)	1.67 ± 3.1	2.51 ± 3.43	1.64 ± 2.8	2.07 ± 3.18

* Standard deviation. AE onset interval denotes days from the vaccination date to the adverse event onset date.

**Table 2:** AEFIs related to delayed recovery after receiving the Janssen COVID-19 vaccine.

SOC	HLT	Non-Recovered Group		HR (95% CI)
		Total (%)	N (%)	
Cardiac disorders	Ischaemic coronary artery disorders	654 (6.72)	654 (6.72)	1.18 (1.09–1.29)
Eye disorders	Ocular disorders NEC	193 (1.98)	193 (1.98)	1.16 (1.01–1.35)
Gastrointestinal disorders	Gastrointestinal and abdominal pains (except oral and throat)	1964 (20.18)	512 (5.26)	1.11 (1.02–1.22)
	Nausea and vomiting symptoms		1858 (19.09)	1.11 (1.05–1.18)
General disorders and administration site conditions	Asthenic conditions	2827 (29.05)	2881 (29.61)	1.06 (1.01–1.12)
	Febrile disorders		2524 (25.94)	1.2 (1.14–1.27)
	Injection site reactions		1090 (11.2)	1.29 (1.21–1.38)
	Pain and discomfort NEC		2540 (26.1)	1.17 (1.12–1.23)
Hepatobiliary disorders	Cholecystitis and cholelithiasis	7 (0.07)	7 (0.07)	2.6 (1.21–5.63)
Immune system disorders	Allergic conditions NEC	207 (2.13)	207 (2.13)	1.27 (1.1–1.46)
Musculoskeletal and connective tissue disorders	Joint-related signs and symptoms	2307 (23.71)	1022 (10.5)	1.12 (1.04–1.19)
	Muscle pains		1006 (10.34)	1.17 (1.09–1.26)
	Musculoskeletal and connective tissue pain and discomfort		1934 (19.87)	1.12 (1.06–1.18)
Nervous system disorders	Auditory nerve disorders	3769 (38.73)	393 (4.04)	1.41 (1.27–1.57)
	Headaches NEC		3526 (36.23)	1.16 (1.11–1.22)
	Sensory abnormalities NEC		335 (3.44)	1.22 (1.09–1.36)
Psychiatric disorders	Cognitive and attention disorders and disturbances NEC	314 (3.23)	117 (1.2)	1.21 (1.01–1.46)
	Disturbances in initiating and maintaining sleep		207 (2.13)	1.19 (1.03–1.37)
Respiratory, thoracic, and mediastinal disorders	Coughing and associated symptoms	367 (3.77)	314 (3.23)	1.17 (1.03–1.32)
	Pharyngeal disorders (except infections and neoplasms)		67 (0.69)	1.34 (1.05–1.72)
Skin and subcutaneous tissue disorders	Dermal and epidermal conditions NEC	1485 (15.26)	1264 (12.99)	1.08 (1.02–1.15)
	Erythemas		417 (4.29)	1.12 (1.01–1.24)

Note: Non-Recovered Group—includes individuals who had not recovered from Janssen COVID-19-vaccine-induced AEs. For 2 weeks, no information on recovery was an exclusion criterion. NEC—not elsewhere classified—a standard abbreviation used to denote miscellaneous terms that do not readily fit into other hierarchical classifications for a particular SOC. The NEC designation is used only with HLTs and HLGTs for grouping purposes. Total—denotes the number of cases for one or more adverse symptoms belonging to each SOC. HR—hazard ratios.

**Table 3:** AEFIs related to delayed recovery after receiving the Moderna COVID-19 vaccine.

SOC	HLT	Non-Recovered Group		HR (95% CI)
		Total (%)	N (%)	
Blood and lymphatic system disorders	Lymphatic system disorders NEC	2943 (4.88)	2943 (4.88)	1.15 (1.11–1.2)
Cardiac disorders	Cardiac signs and symptoms NEC	6622 (10.98)	5455 (9.05)	1.07 (1.04–1.1)
	Ischaemic coronary artery disorders		2016 (3.34)	1.06 (1.01–1.11)
	Left ventricular failures		84 (0.14)	1.39 (1.11–1.73)
	Ventricular arrhythmias and cardiac arrest		252 (0.42)	1.15 (1–1.31)
Ear and labyrinth disorders	Ear disorders NEC	743 (1.23)	743 (1.23)	1.1 (1.02–1.19)
Endocrine disorders	Hypoglycaemic conditions NEC	12 (0.02)	12 (0.02)	2.13 (1.21–3.73)
Eye disorders	Conjunctival infections, irritations, and inflammations	1093 (1.81)	30 (0.05)	1.46 (1.02–2.1)
	Lacrimation disorders		170 (0.28)	1.22 (1.04–1.42)
	Ocular disorders NEC		654 (1.08)	1.14 (1.05–1.24)
	Ocular infections, inflammations, and associated manifestations		347 (0.58)	1.16 (1.04–1.29)
	Visual impairment and blindness (except colour blindness)		285 (0.47)	1.13 (1–1.27)
Gastrointestinal disorders	Diarrhoea (except infective)	8291 (13.75)	2049 (3.4)	1.05 (1–1.1)
	Gastrointestinal and abdominal pains (except oral and throat)		1127 (1.87)	1.07 (1–1.14)
	Gastrointestinal disorders NEC		66 (0.11)	1.29 (1.01–1.65)
	Nausea and vomiting symptoms		7059 (11.71)	1.07 (1.04–1.1)
	Oral soft tissue disorders NEC		76 (0.13)	1.3 (1.03–1.63)
	Oral soft tissue signs and symptoms		2022 (3.35)	1.15 (1.09–1.2)
	Oral soft tissue swelling and oedema		10 (0.02)	2.28 (1.23–4.23)
	Stomatitis and ulceration		183 (0.3)	1.24 (1.06–1.44)
	Tongue signs and symptoms		203 (0.34)	1.21 (1.05–1.39)
General disorders and administration site conditions	Asthenic conditions	16,454 (27.29)	12,452 (20.65)	1.06 (1.03–1.08)
	Death and sudden death		1179 (1.96)	1.57 (1.47–1.67)
	Febrile disorders		9564 (15.86)	1.14 (1.11–1.17)
	General signs and symptoms NEC		9468 (15.7)	1.1 (1.07–1.12)
	Pain and discomfort NEC		11,624 (19.28)	1.16 (1.14–1.19)
Hepatobiliary disorders	Hepatobiliary signs and symptoms	15 (0.02)	15 (0.02)	1.86 (1.11–3.11)
Immune system disorders	Allergic conditions NEC	2035 (3.38)	751 (1.25)	1.25 (1.16–1.34)
	Angio-oedemas		1364 (2.26)	1.1 (1.04–1.16)
Infections and infestations	Clostridia infections	13 (0.02)	13 (0.02)	3.47 (2.02–5.94)
Injury, poisoning and procedural complications	Chest and respiratory tract injuries NEC	96 (0.16)	8 (0.01)	2.42 (1.21–4.85)
	Muscle, tendon, and ligament injuries		88 (0.15)	1.32 (1.07–1.63)
Investigations	Gastrointestinal histopathology procedures	264 (0.44)	9 (0.01)	1.98 (1.04–3.79)
	Musculoskeletal and soft tissue imaging procedures		255 (0.42)	1.23 (1.08–1.4)
Metabolism and nutrition disorders	Fluid intake decreased	18 (0.03)	18 (0.03)	1.83 (1.14–2.94)
Musculoskeletal and connective tissue disorders	Bursal disorders	11,522 (19.11)	49 (0.08)	1.65 (1.25–2.2)
	Joint-related disorders NEC		121 (0.2)	1.56 (1.31–1.87)
	Joint-related signs and symptoms		5216 (8.65)	1.25 (1.21–1.29)
	Muscle pains		3721 (6.17)	1.05 (1.02–1.09)
	Musculoskeletal and connective tissue conditions NEC		844 (1.40)	1.08 (1.01–1.16)
	Musculoskeletal and connective tissue pain and discomfort		9701 (16.09)	1.16 (1.13–1.18)
	Skull and face fractures		7 (0.01)	2.45 (1.14–5.28)
	Soft tissue disorders NEC		171 (0.28)	1.24 (1.07–1.44)
Nervous system disorders	Auditory nerve disorders	14,617 (24.24)	2110 (3.5)	1.49 (1.42–1.56)
	Dementia (except Alzheimer’s type)		23 (0.04)	1.86 (1.22–2.83)
	Headaches NEC		11,704 (19.41)	1.2 (1.17–1.23)
	Neurologic visual problems NEC		722 (1.2)	1.15 (1.06–1.24)
	Neuromuscular disorders NEC		695 (1.15)	1.17 (1.08–1.26)
	Paraesthesia and dysesthesias		908 (1.51)	1.22 (1.15–1.31)
	Paralysis and paresis (except cranial nerve)		200 (0.33)	1.17 (1.02–1.35)
	Sensory abnormalities NEC		1277 (2.12)	1.2 (1.13–1.27)
Psychiatric disorders	Cognitive and attention disorders and disturbances NEC	2483 (4.12)	418 (0.69)	1.29 (1.16–1.42)
	Confusion and disorientation		579 (0.96)	1.15 (1.06–1.25)
	Disturbances in initiating and maintaining sleep		632 (1.05)	1.24 (1.14–1.34)
	Sleep disorders NEC		1168 (1.94)	1.19 (1.12–1.26)
Reproductive system and breast disorders	Erection and ejaculation conditions and disorders	15 (0.02)	15 (0.02)	2.2 (1.32–3.67)
Respiratory, thoracic, and mediastinal disorders	Breathing abnormalities	3293 (5.46)	2595 (4.3)	1.1 (1.06–1.15)
	Coughing and associated symptoms		1344 (2.23)	1.22 (1.15–1.29)
Skin and subcutaneous tissue disorders	Dermal and epidermal conditions NEC	7289 (12.09)	7289 (12.09)	1.07 (1.05–1.1)
Social circumstances	Disability issues	1335 (2.21)	1335 (2.21)	1.08 (1.02–1.14)
Vascular disorders	Bruising, ecchymosis, and purpura	1724 (2.86)	934 (1.55)	1.17 (1.1–1.25)
	Cerebrovascular and spinal vascular disorders NEC		810 (1.34)	1.14 (1.06–1.23)

Note: Non-Recovered Group—includes individuals who had not recovered from Moderna COVID-19-vaccine-induced AEs. For 2 weeks, no information on recovery was an exclusion criterion. NEC—not elsewhere classified—a standard abbreviation used to denote miscellaneous terms that do not readily fit into other hierarchical classifications for a particular SOC. The NEC designation is used only with HLTs and HLGTs for grouping purposes. Total—denotes the number of cases for one or more adverse symptoms belonging to each SOC. HR—hazard ratios.

**Table 4:** AEFIs related to delayed recovery after receiving the Pfizer-BioNTech COVID-19 vaccine.

SOC	HLT	Non-Recovered Group		HR (95% CI)
		Total (%)	N (%)	
Blood and lymphatic system disorders	Lymphatic system disorders NEC	3735 (7.41)	3735 (7.41)	1.25 (1.21–1.29)
Cardiac disorders	Heart failure signs and symptoms	3108 (6.17)	204 (0.4)	1.2 (1.04–1.38)
	Ischaemic coronary artery disorders		2745 (5.45)	1.05 (1.01–1.1)
	Ventricular arrhythmias and cardiac arrest		287 (0.57)	1.34 (1.18–1.52)
Endocrine disorders	Diabetic complications neurological	14 (0.03)	14 (0.03)	1.73 (1.02–2.92)
Eye disorders	Choroid and vitreous haemorrhages and vascular disorders	897 (1.78)	6 (0.01)	3.37 (1.48–7.7)
	Ocular disorders NEC		891 (1.77)	1.13 (1.05–1.21)
Gastrointestinal disorders	Flatulence, bloating, and distension	9051 (17.97)	269 (0.53)	1.13 (1–1.28)
	Gastrointestinal and abdominal pains (except oral and throat)		1505 (2.99)	1.11 (1.05–1.17)
	Gastrointestinal disorders NEC		99 (0.2)	1.3 (1.07–1.59)
	Gastrointestinal dyskinetic disorders		35 (0.07)	1.54 (1.1–2.15)
	Nausea and vomiting symptoms		6933 (13.76)	1.04 (1.01–1.07)
	Oral soft tissue signs and symptoms		2635 (5.23)	1.1 (1.06–1.15)
	Tongue signs and symptoms		251 (0.5)	1.14 (1–1.29)
General disorders and administration site conditions	Death and sudden death	16,022 (31.8)	848 (1.68)	1.39 (1.3–1.5)
	Feelings and sensations NEC		3245 (6.44)	1.08 (1.04–1.12)
	Gait disturbances		930 (1.85)	1.07 (1–1.15)
	General signs and symptoms NEC		6709 (13.32)	1.07 (1.04–1.1)
	Injection site reactions		4516 (8.96)	1.1 (1.06–1.13)
	Pain and discomfort NEC		9810 (19.47)	1.08 (1.05–1.11)
	Vaccination site reactions		1628 (3.23)	1.88 (1.79–1.98)
Immune system disorders	Allergic conditions NEC	716 (1.42)	716 (1.42)	1.3 (1.21–1.4)
Infections and infestations	Cardiac infections	6 (0.01)	6 (0.01)	2.72 (1.2–6.14)
Injury, poisoning and procedural complications	Medication errors, product use errors and issues NEC	491 (0.97)	110 (0.22)	1.51 (1.17–1.95)
	Off label uses		134 (0.27)	2.16 (1.71–2.74)
	Overdoses NEC		14 (0.03)	1.84 (1.08–3.11)
	Product administration errors and issues		507 (1.01)	1.26 (1.15–1.38)
	Radiation injuries		15 (0.03)	1.67 (1–2.76)
Investigations	Heart rate and pulse investigations	3858 (7.66)	1458 (2.89)	1.1 (1.04–1.17)
	Physical examination procedures and organ system status		2411 (4.79)	1.13 (1.09–1.18)
	Vascular tests NEC (include blood pressure)		850 (1.69)	1.1 (1.02–1.18)
Musculoskeletal and connective tissue disorders	Joint-related disorders NEC	9847 (19.55)	102 (0.2)	1.26 (1.03–1.53)
	Joint-related signs and symptoms		5150 (10.22)	1.12 (1.09–1.16)
	Muscle pains		3822 (7.59)	1.08 (1.04–1.12)
	Musculoskeletal and connective tissue conditions NEC		842 (1.67)	1.09 (1.02–1.17)
	Musculoskeletal and connective tissue pain and discomfort		8048 (15.98)	1.19 (1.16–1.22)
Nervous system disorders	Auditory nerve disorders	15,176 (30.13)	2701 (5.36)	1.34 (1.29–1.4)
	Dementia (except Alzheimer’s type)		13 (0.03)	2.13 (1.23–3.67)
	Headaches NEC		11,537 (22.9)	1.13 (1.11–1.16)
	Neurologic visual problems NEC		917 (1.82)	1.08 (1.01–1.15)
	Neuromuscular disorders NEC		806 (1.6)	1.08 (1–1.16)
	Paraesthesia and dysesthesias		981 (1.95)	1.1 (1.03–1.17)
	Peripheral neuropathies NEC		205 (0.41)	1.21 (1.05–1.39)
	Sensory abnormalities NEC		1671 (3.32)	1.18 (1.12–1.24)
Psychiatric disorders	Cognitive and attention disorders and disturbances NEC	1490 (2.96)	478 (0.95)	1.16 (1.05–1.27)
	Communications disorders		79 (0.16)	1.27 (1.01–1.58)
	Disturbances in initiating and maintaining sleep		792 (1.57)	1.17 (1.08–1.25)
	Emotional and mood disturbances NEC		283 (0.56)	1.13 (1–1.28)
	Parasomnias		53 (0.11)	1.34 (1.02–1.76)
Respiratory, thoracic, and mediastinal disorders	Breathing abnormalities	3346 (6.64)	3097 (6.15)	1.12 (1.08–1.17)
	Pharyngeal disorders (except infections and neoplasms)		419 (0.83)	1.22 (1.1–1.34)
Skin and subcutaneous tissue disorders	Dermal and epidermal conditions NEC	7584 (15.05)	5694 (11.3)	1.2 (1.16–1.23)
	Pruritus NEC		2898 (5.75)	1.07 (1.03–1.12)
Vascular disorders	Cerebrovascular and spinal vascular disorders NEC	917 (1.82)	917 (1.82)	1.1 (1.03–1.17)

Note: Non-Recovered Group—includes individuals who had not recovered from Pfizer-BioNTech COVID-19-vaccine-induced AEs. For 2 weeks, no information on recovery was an exclusion criterion. NEC—not elsewhere classified—a standard abbreviation used to denote miscellaneous terms that do not readily fit into other hierarchical classifications for a particular SOC. The NEC designation is used only with HLTs and HLGTs for grouping purposes. Total—denotes the number of cases for one or more adverse symptoms belonging to each SOC. HR—hazard ratios.

## Data Availability

The raw data used in the current study are available in the VAERS (https://vaers.hhs.gov/data/datasets.html, accessed on: 6 August 2021). All data generated in this study are available on request from the corresponding author.

## Supplementary Materials

The following supporting information can be downloaded at: https://www.mdpi.com/article/10.3390/vaccines10020284/s1. Table S1: Baseline characteristics of individuals vaccinated with the Janssen COVID-19 vaccine before and after PSM, Table S2: Baseline characteristics of individuals vaccinated with the Moderna COVID-19 vaccine before and after PSM, Table S3: Baseline characteristics of individuals vaccinated with the Pfizer-BioNTech COVID-19 vaccine before and after PSM, Figure S1: AE hazard ratios after Janssen COVID-19 vaccination estimated from a multivariate Cox model, Figure S2: AE hazard ratios after Moderna COVID-19 vaccination estimated from a multivariate Cox model, Figure S3: AE hazard ratios after Pfizer-BioNTech COVID-19 vaccination estimated from a multivariate Cox model.
