# Supplementary material for: Propensity-Score-Matched Evaluation of Adverse Events Affecting Recovery after COVID-19 Vaccination: On Adenovirus and mRNA Vaccines
Source: Vaccines (Basel). 2022 Feb 13;10(2):284. doi: 10.3390/vaccines10020284 (PMC8875227; doi:10.3390/vaccines10020284)
Supplement: Supplementary file 1 [file vaccines-10-00284-s001.zip › vaccines-1565536-supplementary.pdf]

Propensity Score-Matched Evaluation of Adverse Events Affecting Recovery After COVID-19 Vaccination: On Adenovirus and mRNA Vaccines

Chang-Sik Son *, Sang-Hyeon Jin and Won-Seok Kang *

Division of Intelligent Robot, Daegu Gyeongbuk Institute of Science and Technology (DGIST), Daegu 42988, South Korea; jinjinsh@dgist.ac.kr (S.H.J.)
* Correspondence: changsikson@dgist.ac.kr (C.S.S.); wskang@dgist.ac.kr (W.S.K.)

Supplementary information

Supplementary Table S1. Baseline characteristics of individuals vaccinated with the Janssen COVID-19 vaccine before and after PSM.

Supplementary Table S2. Baseline characteristics of individuals vaccinated with the Moderna COVID-19 vaccine before and after PSM.

Supplementary Table S3. Baseline characteristics of individuals vaccinated with the Pfizer-BioNTech COVID-19 vaccine before and after PSM.

Supplementary Figure S1. AE hazard ratios after Janssen COVID-19 vaccination estimated from a multivariate Cox model.

Supplementary Figure S2. AE hazard ratios after Moderna COVID-19 vaccination estimated from a multivariate Cox model.

Supplementary Figure S3. AE hazard ratios after Pfizer-BioNTech COVID-19 vaccination estimated from a multivariate Cox model.

Table S1. Baseline characteristics of individuals vaccinated with the Janssen COVID-19 vaccine before and after PSM.

Variable	Before PSM				After PSM			
	No Recovery	Recovery	ASD	P-value	No Recovery	Recovery	ASD	P-value
Total, N	10,274	11,763			9,731	9,731		
Age				0.000				0.949
20-29, N (%)	1,443 (14.05)	2,790 (23.72)	0.249		1,443 (14.83)	1,421 (14.6)	0.006	
30-39, N (%)	2,115 (20.59)	2,715 (23.08)	0.060		2,107 (21.65)	2,097 (21.55)	0.002	
40-49, N (%)	2,178 (21.2)	2,146 (18.24)	0.074		2,084 (21.42)	2,106 (21.64)	0.006	
50-59, N (%)	2,342 (22.8)	2,278 (19.37)	0.084		2,220 (22.81)	2,277 (23.4)	0.014	
60-69, N (%)	1,545 (15.04)	1,382 (11.75)	0.097		1,413 (14.52)	1,380 (14.18)	0.010	
70-79, N (%)	462 (4.5)	349 (2.97)	0.081		359 (3.69)	347 (3.57)	0.007	
≥80, N (%)	189 (1.84)	103 (0.88)	0.083		105 (1.08)	103 (1.06)	0.002	
Gender				0.000				0.783
Unknown, N (%)	13 (0.13)	21 (0.18)	0.013		13 (0.13)	12 (0.12)	0.003	
Male, N (%)	3,174 (30.89)	4,264 (36.25)	0.114		3,056 (31.4)	3,013 (30.96)	0.010	
Female, N (%)	7,087 (68.98)	7,478 (63.57)	0.115		6,662 (68.46)	6,706 (68.91)	0.010	
Underlying disability								
Obesity, N (%)	158 (1.54)	122 (1.04)	0.044	0.001	135 (1.39)	118 (1.21)	0.015	0.311
Hypertension, N (%)	814 (7.92)	623 (5.3)	0.106	0.000	646 (6.64)	611 (6.28)	0.015	0.321
Diabetes, N (%)	467 (4.55)	325 (2.76)	0.095	0.000	356 (3.66)	324 (3.33)	0.018	0.226
Atrial fibrillation, N (%)	15 (0.15)	19 (0.16)	0.004	0.904	14 (0.14)	12 (0.12)	0.006	0.844
Heart failure, N (%)	82 (0.8)	44 (0.37)	0.056	0.000	55 (0.57)	44 (0.45)	0.016	0.314
Kidney disease, N (%)	46 (0.45)	32 (0.27)	0.029	0.038	34 (0.35)	31 (0.32)	0.005	0.804
Pulmonary disease, N (%)	135 (1.31)	52 (0.44)	0.094	0.000	55 (0.57)	52 (0.53)	0.004	0.846
Asthma, N (%)	578 (5.63)	483 (4.11)	0.071	0.000	491 (5.05)	479 (4.92)	0.006	0.717
Stroke, N (%)	18 (0.18)	18 (0.15)	0.005	0.811	16 (0.16)	17 (0.17)	0.002	1.000
Cancers, N (%)	111 (1.08)	89 (0.76)	0.034	0.014	90 (0.92)	87 (0.89)	0.003	0.880
Allergies, N (%)	296 (2.88)	263 (2.24)	0.041	0.003	256 (2.63)	249 (2.56)	0.005	0.787

Note: ASD, absolute standardised difference; P-values were calculated using Pearson’s chi-squared tests.

Table S2. Baseline characteristics of individuals vaccinated with the Moderna COVID-19 vaccine before and after PSM.

Variable	Before PSM				After PSM			
	No Recovery	Recovery	ASD	P-value	No Recovery	Recovery	ASD	P-value
Total, N	63,170	63,668			60,290	60,290		
Age				0.000				0.206
20-29, N (%)	6,079 (9.62)	7,555 (11.87)	0.072		6,073 (10.07)	6,006 (9.96)	0.004	
30-39, N (%)	11,367 (17.99)	11,189 (17.57)	0.011		10,673 (17.7)	10,513 (17.44)	0.007	
40-49, N (%)	11,949 (18.92)	11,034 (17.33)	0.041		10,802 (17.92)	10,902 (18.08)	0.004	
50-59, N (%)	11,629 (18.41)	11,670 (18.33)	0.002		11,334 (18.8)	11,314 (18.77)	0.001	
60-69, N (%)	11,762 (18.62)	12,346 (19.39)	0.020		11,501 (19.08)	11,695 (19.4)	0.008	
70-79, N (%)	7,559 (11.97)	7,481 (11.75)	0.007		7,377 (12.24)	7,473 (12.4)	0.005	
≥80, N (%)	2,825 (4.47)	2,393 (3.76)	0.036		2,530 (4.2)	2,387 (3.96)	0.012	
Gender				0.000				0.652
Unknown, N (%)	85 (0.13)	140 (0.22)	0.020		82 (0.14)	83 (0.14)	0.000	
Male, N (%)	14,122 (22.36)	16,039 (25.19)	0.067		13,976 (23.18)	13,841 (22.96)	0.005	
Female, N (%)	48,963 (77.51)	47,489 (74.59)	0.068		46,232 (76.68)	46,366 (76.9)	0.005	
Underlying disability								
Obesity, N (%)	1,119 (1.77)	908 (1.43)	0.028	0.000	939 (1.56)	902 (1.5)	0.005	0.398
Hypertension, N (%)	6,416 (10.16)	5,732 (9.0)	0.039	0.000	5,724 (9.49)	5,725 (9.5)	0.000	1.000
Diabetes, N (%)	3,660 (5.79)	3,239 (5.09)	0.031	0.000	3,213 (5.33)	3,234 (5.36)	0.002	0.798
Atrial fibrillation, N (%)	184 (0.29)	180 (0.28)	0.002	0.816	172 (0.29)	170 (0.28)	0.001	0.957
Heart failure, N (%)	598 (0.95)	475 (0.75)	0.022	0.000	495 (0.82)	472 (0.78)	0.004	0.478
Kidney disease, N (%)	411 (0.65)	371 (0.58)	0.009	0.131	374 (0.62)	361 (0.6)	0.003	0.657
Pulmonary disease, N (%)	779 (1.23)	617 (0.97)	0.025	0.000	621 (1.03)	612 (1.02)	0.001	0.819
Asthma, N (%)	3,401 (5.38)	3,179 (4.99)	0.018	0.002	3,102 (5.15)	3,155 (5.23)	0.004	0.500
Stroke, N (%)	160 (0.25)	125 (0.2)	0.012	0.037	139 (0.23)	124 (0.21)	0.005	0.387
Cancers, N (%)	926 (1.47)	842 (1.32)	0.012	0.031	853 (1.41)	840 (1.39)	0.002	0.769
Allergies, N (%)	1,774 (2.81)	1,482 (2.33)	0.030	0.000	1,452 (2.41)	1,478 (2.45)	0.003	0.640

Note: ASD, absolute standardised difference; P-values were calculated using Pearson's chi-squared tests.

Table S3. Baseline characteristics of individuals vaccinated with the Pfizer-BioNTech COVID-19 vaccine before and after PSM.

Variable	Before PSM				After PSM			
	No Recovery	Recovery	ASD	P-value	No Recovery	Recovery	ASD	P-value
Total, N	54,729	53,390			50,376	50,376		
Age				0.000				0.979
20-29, N (%)	5,610 (10.25)	7,817 (14.64)	0.133		5,600 (11.12)	5,602 (11.12)	0.000	
30-39, N (%)	10,213 (18.66)	10,743 (20.12)	0.037		10,210 (20.27)	10,186 (20.22)	0.001	
40-49, N (%)	11,178 (20.42)	10,239 (19.18)	0.031		10,199 (20.25)	10,186 (20.22)	0.001	
50-59, N (%)	11,108 (20.3)	9,921 (18.58)	0.043		9,910 (19.67)	9,885 (19.62)	0.001	
60-69, N (%)	9,278 (16.95)	8,516 (15.95)	0.027		8,408 (16.69)	8,385 (16.64)	0.001	
70-79, N (%)	5,150 (9.41)	4,489 (8.41)	0.035		4,376 (8.69)	4,471 (8.88)	0.007	
≥80, N (%)	2,192 (4.01)	1,665 (3.12)	0.048		1,673 (3.32)	1,661 (3.3)	0.001	
Gender				0.000				0.068
Unknown, N (%)	157 (0.29)	302 (0.57)	0.043		156 (0.31)	155 (0.31)	0.000	
Male, N (%)	14,809 (27.06)	13,872 (25.98)	0.024		12,768 (25.35)	12,450 (24.71)	0.015	
Female, N (%)	39,763 (72.65)	39,216 (73.45)	0.018		37,452 (74.34)	37,771 (74.98)	0.015	
Underlying disability								
Obesity, N (%)	692 (1.26)	686 (1.28)	0.002	0.785	655 (1.3)	674 (1.34)	0.003	0.619
Hypertension, N (%)	3,945 (7.21)	3,815 (7.15)	0.002	0.698	3,827 (7.6)	3,674 (7.29)	0.012	0.068
Diabetes, N (%)	2,321 (4.24)	1,967 (3.68)	0.029	0.000	19,98 (3.97)	1,948 (3.87)	0.005	0.426
Atrial fibrillation, N (%)	152 (0.28)	135 (0.25)	0.005	0.462	136 (0.27)	133 (0.26)	0.001	0.903
Heart failure, N (%)	384 (0.7)	292 (0.55)	0.020	0.001	308 (0.61)	287 (0.57)	0.005	0.411
Kidney disease, N (%)	260 (0.48)	264 (0.49)	0.003	0.678	250 (0.5)	245 (0.49)	0.001	0.857
Pulmonary disease, N (%)	469 (0.86)	329 (0.62)	0.028	0.000	301 (0.6)	326 (0.65)	0.006	0.336
Asthma, N (%)	2,330 (4.26)	2,550 (4.78)	0.025	0.000	2,305 (4.58)	2,348 (4.66)	0.004	0.528
Stroke, N (%)	118 (0.22)	116 (0.22)	0.000	0.995	114 (0.23)	108 (0.21)	0.003	0.737
Cancers, N (%)	625 (1.14)	596 (1.12)	0.002	0.711	585 (1.16)	583 (1.16)	0.000	0.977
Allergies, N (%)	1,304 (2.38)	1,253 (2.35)	0.002	0.714	1,227 (2.44)	1,207 (2.4)	0.003	0.697

Note: ASD, absolute standardised difference; P-values were calculated using Pearson’s chi-squared test.

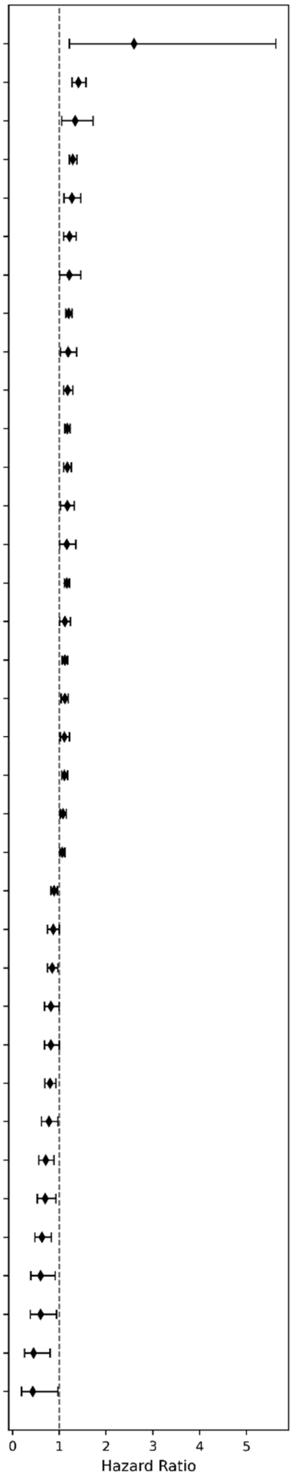

	N	HR	95% CI	P
cholecystitis and cholelithiasis	7	2.6	(1.21, 5.63)	<.05
auditory nerve disorders	393	1.41	(1.27, 1.57)	<.0001
pharyngeal disorders (excl infections and neoplasms)	67	1.34	(1.05, 1.72)	<.05
injection site reactions	1090	1.29	(1.21, 1.38)	<.0001
allergic conditions nec	207	1.27	(1.10, 1.46)	<.001
sensory abnormalities nec	335	1.22	(1.09, 1.36)	<.001
cognitive and attention disorders and disturbances nec	117	1.21	(1.01, 1.46)	<.05
febrile disorders	2524	1.2	(1.14, 1.27)	<.0001
disturbances in initiating and maintaining sleep	207	1.19	(1.03, 1.37)	<.05
ischaemic coronary artery disorders	654	1.18	(1.09, 1.29)	<.001
pain and discomfort nec	2540	1.17	(1.12, 1.23)	<.0001
muscle pains	1006	1.17	(1.09, 1.26)	<.0001
coughing and associated symptoms	314	1.17	(1.03, 1.32)	<.05
ocular disorders nec	193	1.16	(1.01, 1.35)	<.05
headaches nec	3526	1.16	(1.11, 1.22)	<.0001
erythemas	417	1.12	(1.01, 1.24)	<.05
musculoskeletal and connective tissue pain and discomfort	1934	1.12	(1.06, 1.18)	<.0001
joint related signs and symptoms	1022	1.12	(1.04, 1.19)	<.01
gastrointestinal and abdominal pains (excl oral and throat)	512	1.11	(1.02, 1.22)	<.05
nausea and vomiting symptoms	1858	1.11	(1.05, 1.18)	<.001
dermal and epidermal conditions nec	1264	1.08	(1.02, 1.15)	<.05
asthenic conditions	2881	1.06	(1.01, 1.12)	<.05
imaging procedures nec	789	0.89	(0.82, 0.96)	<.01
neurological signs and symptoms nec	205	0.87	(0.75, 1.00)	<.05
virus identification and serology	331	0.85	(0.75, 0.97)	<.05
peripheral embolism and thrombosis	143	0.82	(0.68, 0.99)	<.05
cardiac function diagnostic procedures	123	0.82	(0.68, 1.00)	<.05
disturbances in consciousness nec	196	0.8	(0.69, 0.93)	<.01
white blood cell analyses	118	0.78	(0.62, 0.97)	<.05
haemorrhages nec	75	0.71	(0.56, 0.89)	<.01
peripheral vasoconstriction, necrosis and vascular insufficiency	52	0.7	(0.53, 0.93)	<.05
viral lower respiratory tract infections	66	0.63	(0.48, 0.83)	<.001
exposures associated with pregnancy, delivery and lactation	22	0.6	(0.39, 0.91)	<.05
cerebrovascular and spinal necrosis and vascular insufficiency	20	0.6	(0.38, 0.94)	<.05
renal disorders nec	12	0.45	(0.26, 0.80)	<.01
abdominal and gastrointestinal infections	6	0.43	(0.19, 0.97)	<.05

Figure S1. AE hazard ratios after Janssen COVID-19 vaccination estimated from a multivariate Cox model.

Note: this supplementary result shows 36 Janssen vaccine-induced AEs with a statistically significant difference ($p < 0.05$) when 91 adverse symptoms associated with the risk of developing an AE, estimated with a univariate Cox proportional hazard regression analysis, were included in the multivariate Cox model for adjustment. N denotes the number of adverse cases in the non-recovered group; HR, hazard ratios; CI, confidence intervals.

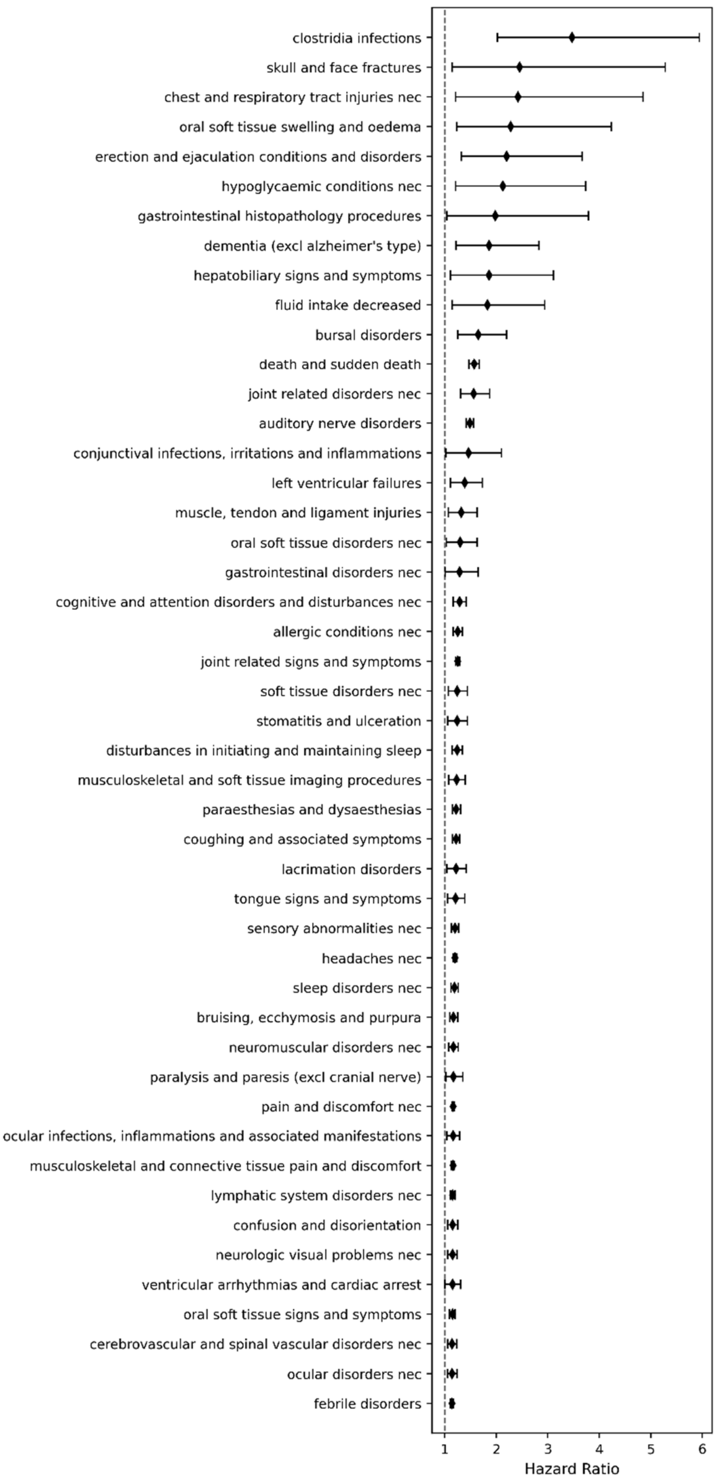

	N	HR	95% CI	P
clostridia infections	13	3.47	(2.02, 5.94)	<.0001
skull and face fractures	7	2.45	(1.14, 5.28)	<.05
chest and respiratory tract injuries nec	8	2.42	(1.21, 4.85)	<.05
oral soft tissue swelling and oedema	10	2.28	(1.23, 4.23)	<.01
erection and ejaculation conditions and disorders	15	2.2	(1.32, 3.67)	<.01
hypoglycaemic conditions nec	12	2.13	(1.21, 3.73)	<.01
gastrointestinal histopathology procedures	9	1.98	(1.04, 3.79)	<.05
dementia (excl alzheimer's type)	23	1.86	(1.22, 2.83)	<.01
hepatobiliary signs and symptoms	15	1.86	(1.11, 3.11)	<.05
fluid intake decreased	18	1.83	(1.14, 2.94)	<.05
bursal disorders	49	1.65	(1.25, 2.20)	<.001
death and sudden death	1179	1.57	(1.47, 1.67)	<.0001
joint related disorders nec	121	1.56	(1.31, 1.87)	<.0001
auditory nerve disorders	2110	1.49	(1.42, 1.56)	<.0001
conjunctival infections, irritations and inflammations	30	1.46	(1.02, 2.10)	<.05
left ventricular failures	84	1.39	(1.11, 1.73)	<.01
muscle, tendon and ligament injuries	88	1.32	(1.07, 1.63)	<.05
oral soft tissue disorders nec	76	1.3	(1.03, 1.63)	<.05
gastrointestinal disorders nec	66	1.29	(1.01, 1.65)	<.05
cognitive and attention disorders and disturbances nec	418	1.29	(1.16, 1.42)	<.0001
allergic conditions nec	751	1.25	(1.16, 1.34)	<.0001
joint related signs and symptoms	5216	1.25	(1.21, 1.29)	<.0001
soft tissue disorders nec	171	1.24	(1.07, 1.44)	<.01
stomatitis and ulceration	183	1.24	(1.06, 1.44)	<.01
disturbances in initiating and maintaining sleep	632	1.24	(1.14, 1.34)	<.0001
musculoskeletal and soft tissue imaging procedures	255	1.23	(1.08, 1.40)	<.01
paraesthesias and dysaesthesias	908	1.22	(1.15, 1.31)	<.0001
coughing and associated symptoms	1344	1.22	(1.15, 1.29)	<.0001
lacrimation disorders	170	1.22	(1.04, 1.42)	<.05
tongue signs and symptoms	203	1.21	(1.05, 1.39)	<.01
sensory abnormalities nec	1277	1.2	(1.13, 1.27)	<.0001
headaches nec	11704	1.2	(1.17, 1.23)	<.0001
sleep disorders nec	1168	1.19	(1.12, 1.26)	<.0001
bruising, ecchymosis and purpura	934	1.17	(1.10, 1.25)	<.0001
neuromuscular disorders nec	695	1.17	(1.08, 1.26)	<.0001
paralysis and paresis (excl cranial nerve)	200	1.17	(1.02, 1.35)	<.05
pain and discomfort nec	11624	1.16	(1.14, 1.19)	<.0001
ocular infections, inflammations and associated manifestations	347	1.16	(1.04, 1.29)	<.01
musculoskeletal and connective tissue pain and discomfort	9701	1.16	(1.13, 1.18)	<.0001
lymphatic system disorders nec	2943	1.15	(1.11, 1.20)	<.0001
confusion and disorientation	579	1.15	(1.06, 1.25)	<.01
neurologic visual problems nec	722	1.15	(1.06, 1.24)	<.001
ventricular arrhythmias and cardiac arrest	252	1.15	(1.00, 1.31)	<.05
oral soft tissue signs and symptoms	2022	1.15	(1.09, 1.20)	<.0001
cerebrovascular and spinal vascular disorders nec	810	1.14	(1.06, 1.23)	<.001
ocular disorders nec	654	1.14	(1.05, 1.24)	<.01
febrile disorders	9654	1.14	(1.11, 1.17)	<.0001

Figure S2. AE hazard ratios after Moderna COVID-19 vaccination estimated from a multivariate Cox model.

Note: this supplementary result shows 94 Moderna vaccine-induced AEs with a statistically significant difference ($p < 0.05$) when 193 adverse symptoms associated with the risk of developing an AE, estimated with a univariate Cox proportional hazard regression analysis, were included in the multivariate Cox model for adjustment. N denotes the number of adverse cases in the non-recovered group; HR, hazard ratios; CI, confidence intervals.

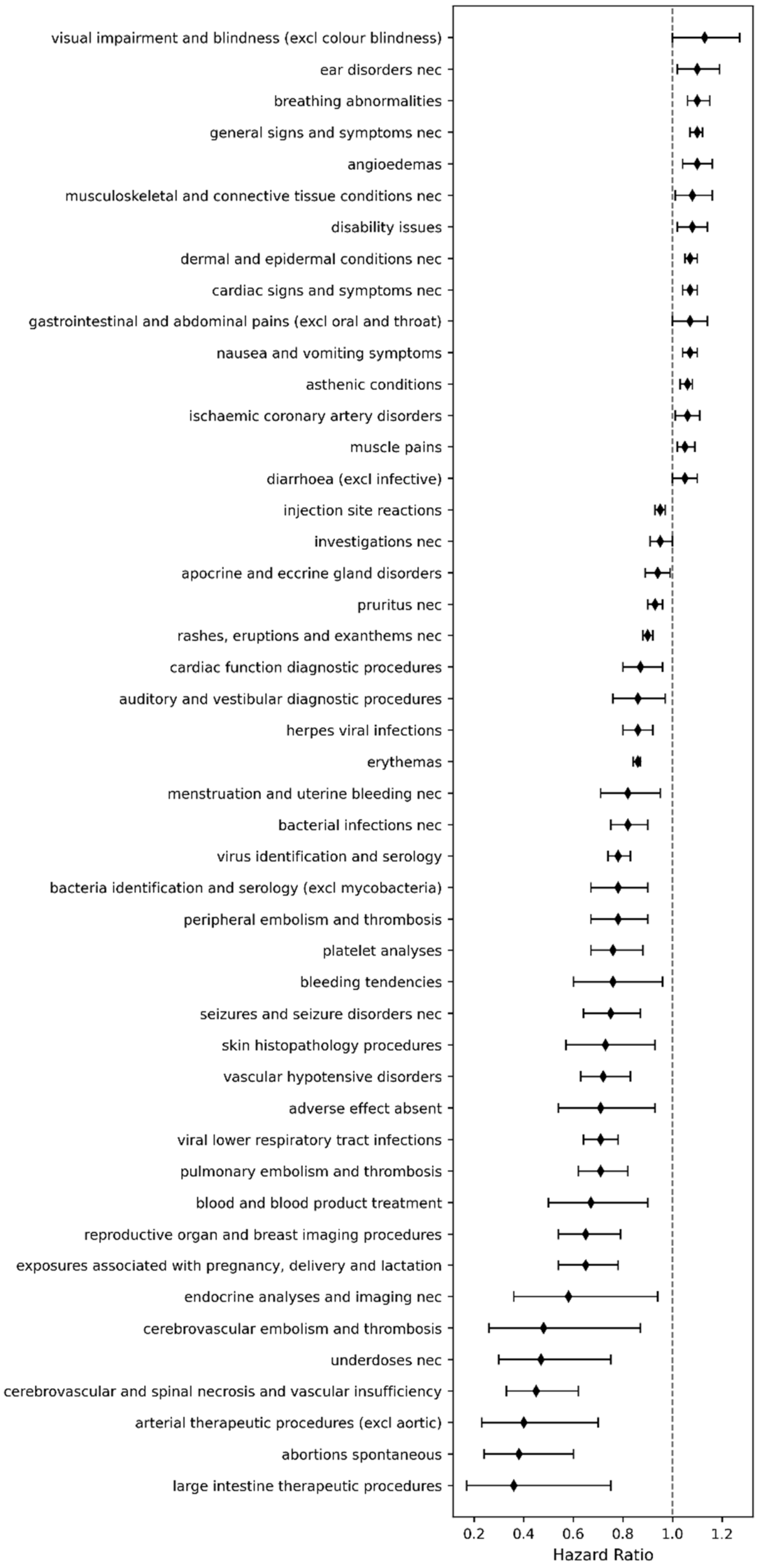

	N	HR	95% CI	P
visual impairment and blindness (excl colour blindness)	285	1.13	(1.00, 1.27)	<.05
ear disorders nec	743	1.1	(1.02, 1.19)	<.05
breathing abnormalities	2595	1.1	(1.06, 1.15)	<.0001
general signs and symptoms nec	9468	1.1	(1.07, 1.12)	<.0001
angioedemas	1364	1.1	(1.04, 1.16)	<.01
musculoskeletal and connective tissue conditions nec	844	1.08	(1.01, 1.16)	<.05
disability issues	1335	1.08	(1.02, 1.14)	<.01
dermal and epidermal conditions nec	7289	1.07	(1.05, 1.10)	<.0001
cardiac signs and symptoms nec	5455	1.07	(1.04, 1.10)	<.0001
gastrointestinal and abdominal pains (excl oral and throat)	1127	1.07	(1.00, 1.14)	<.05
nausea and vomiting symptoms	7059	1.07	(1.04, 1.10)	<.0001
asthenic conditions	12452	1.06	(1.03, 1.08)	<.0001
ischaemic coronary artery disorders	2016	1.06	(1.01, 1.11)	<.05
muscle pains	3721	1.05	(1.02, 1.09)	<.01
diarrhoea (excl infective)	2049	1.05	(1.00, 1.10)	<.05
injection site reactions	19747	0.95	(0.93, 0.97)	<.0001
investigations nec	2481	0.95	(0.91, 1.00)	<.05
apocrine and eccrine gland disorders	1706	0.94	(0.89, 0.99)	<.05
pruritus nec	5462	0.93	(0.90, 0.96)	<.0001
rashes, eruptions and exanthems nec	8906	0.9	(0.88, 0.92)	<.0001
cardiac function diagnostic procedures	576	0.87	(0.80, 0.96)	<.01
auditory and vestibular diagnostic procedures	354	0.86	(0.76, 0.97)	<.05
herpes viral infections	883	0.86	(0.80, 0.92)	<.0001
erythemas	14562	0.86	(0.84, 0.87)	<.0001
menstruation and uterine bleeding nec	201	0.82	(0.71, 0.95)	<.05
bacterial infections nec	473	0.82	(0.75, 0.90)	<.0001
virus identification and serology	1719	0.78	(0.74, 0.83)	<.0001
bacteria identification and serology (excl mycobacteria)	181	0.78	(0.67, 0.90)	<.01
peripheral embolism and thrombosis	196	0.78	(0.67, 0.90)	<.001
platelet analyses	230	0.76	(0.67, 0.88)	<.0001
bleeding tendencies	74	0.76	(0.60, 0.96)	<.05
seizures and seizure disorders nec	169	0.75	(0.64, 0.87)	<.001
skin histopathology procedures	64	0.73	(0.57, 0.93)	<.05
vascular hypotensive disorders	211	0.72	(0.63, 0.83)	<.0001
adverse effect absent	52	0.71	(0.54, 0.93)	<.05
viral lower respiratory tract infections	422	0.71	(0.64, 0.78)	<.0001
pulmonary embolism and thrombosis	232	0.71	(0.62, 0.82)	<.0001
blood and blood product treatment	48	0.67	(0.50, 0.90)	<.01
reproductive organ and breast imaging procedures	114	0.65	(0.54, 0.79)	<.0001
exposures associated with pregnancy, delivery and lactation	137	0.65	(0.54, 0.78)	<.0001
endocrine analyses and imaging nec	17	0.58	(0.36, 0.94)	<.05
cerebrovascular embolism and thrombosis	11	0.48	(0.26, 0.87)	<.05
underdoses nec	20	0.47	(0.30, 0.75)	<.01
cerebrovascular and spinal necrosis and vascular insufficiency	42	0.45	(0.33, 0.62)	<.0001
arterial therapeutic procedures (excl aortic)	13	0.4	(0.23, 0.70)	<.01
abortions spontaneous	22	0.38	(0.24, 0.60)	<.0001
large intestine therapeutic procedures	10	0.36	(0.17, 0.75)	<.01

Figure S2. *Cont.*

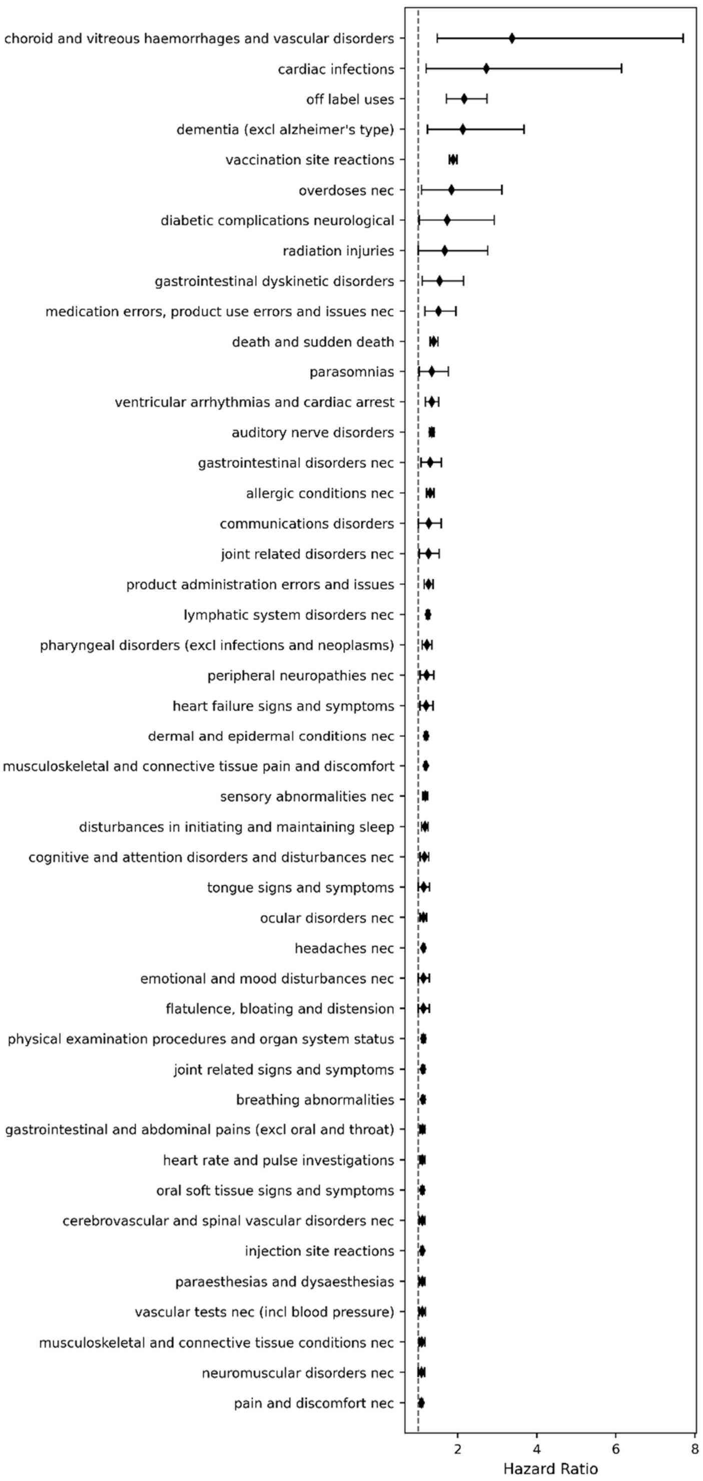

	N	HR	95% CI	P
choroid and vitreous haemorrhages and vascular disorders	6	3.37	(1.48, 7.70)	<.01
cardiac infections	6	2.72	(1.20, 6.14)	<.05
off label uses	134	2.16	(1.71, 2.74)	<.0001
dementia (excl alzheimer's type)	13	2.13	(1.23, 3.67)	<.01
vaccination site reactions	1628	1.88	(1.79, 1.98)	<.0001
overdoses nec	14	1.84	(1.08, 3.11)	<.05
diabetic complications neurological	14	1.73	(1.02, 2.92)	<.05
radiation injuries	15	1.67	(1.00, 2.76)	<.05
gastrointestinal dyskinetic disorders	35	1.54	(1.10, 2.15)	<.05
medication errors, product use errors and issues nec	110	1.51	(1.17, 1.95)	<.01
death and sudden death	848	1.39	(1.30, 1.50)	<.0001
parasomnias	53	1.34	(1.02, 1.76)	<.05
ventricular arrhythmias and cardiac arrest	287	1.34	(1.18, 1.52)	<.0001
auditory nerve disorders	2701	1.34	(1.29, 1.40)	<.0001
gastrointestinal disorders nec	99	1.3	(1.07, 1.59)	<.01
allergic conditions nec	716	1.3	(1.21, 1.40)	<.0001
communications disorders	79	1.27	(1.01, 1.58)	<.05
joint related disorders nec	102	1.26	(1.03, 1.53)	<.05
product administration errors and issues	507	1.26	(1.15, 1.38)	<.0001
lymphatic system disorders nec	3735	1.25	(1.21, 1.29)	<.0001
pharyngeal disorders (excl infections and neoplasms)	419	1.22	(1.10, 1.34)	<.001
peripheral neuropathies nec	205	1.21	(1.05, 1.39)	<.01
heart failure signs and symptoms	204	1.2	(1.04, 1.38)	<.01
dermal and epidermal conditions nec	5694	1.2	(1.16, 1.23)	<.0001
musculoskeletal and connective tissue pain and discomfort	8048	1.19	(1.16, 1.22)	<.0001
sensory abnormalities nec	1671	1.18	(1.12, 1.24)	<.0001
disturbances in initiating and maintaining sleep	792	1.17	(1.08, 1.25)	<.0001
cognitive and attention disorders and disturbances nec	478	1.16	(1.05, 1.27)	<.01
tongue signs and symptoms	251	1.14	(1.00, 1.29)	<.05
ocular disorders nec	891	1.13	(1.05, 1.21)	<.01
headaches nec	11537	1.13	(1.11, 1.16)	<.0001
emotional and mood disturbances nec	283	1.13	(1.00, 1.28)	<.05
flatulence, bloating and distension	269	1.13	(1.00, 1.28)	<.05
physical examination procedures and organ system status	2411	1.13	(1.09, 1.18)	<.0001
joint related signs and symptoms	5150	1.12	(1.09, 1.16)	<.0001
breathing abnormalities	3097	1.12	(1.08, 1.17)	<.0001
gastrointestinal and abdominal pains (excl oral and throat)	1505	1.11	(1.05, 1.17)	<.001
heart rate and pulse investigations	1458	1.1	(1.04, 1.17)	<.001
oral soft tissue signs and symptoms	2635	1.1	(1.06, 1.15)	<.0001
cerebrovascular and spinal vascular disorders nec	917	1.1	(1.03, 1.17)	<.01
injection site reactions	4516	1.1	(1.06, 1.13)	<.0001
paraesthesias and dysaesthesias	981	1.1	(1.03, 1.17)	<.01
vascular tests nec (incl blood pressure)	850	1.1	(1.02, 1.18)	<.05
musculoskeletal and connective tissue conditions nec	842	1.09	(1.02, 1.17)	<.05
neuromuscular disorders nec	806	1.08	(1.00, 1.16)	<.05
pain and discomfort nec	9810	1.08	(1.05, 1.11)	<.0001

Figure S3. AE hazard ratios after Pfizer-BioNTech COVID-19 vaccination estimated from a multivariate Cox model.

Note: this supplementary result shows 92 Pfizer-BioNTech vaccine-induced AEs with a statistically significant difference ($p < 0.05$) when 208 adverse symptoms associated with the risk of developing an AE, estimated with a univariate Cox proportional hazard regression analysis, were included in the multivariate Cox model for adjustment. N denotes the number of adverse cases in the non-recovered group; HR, hazard ratios; CI, confidence intervals.

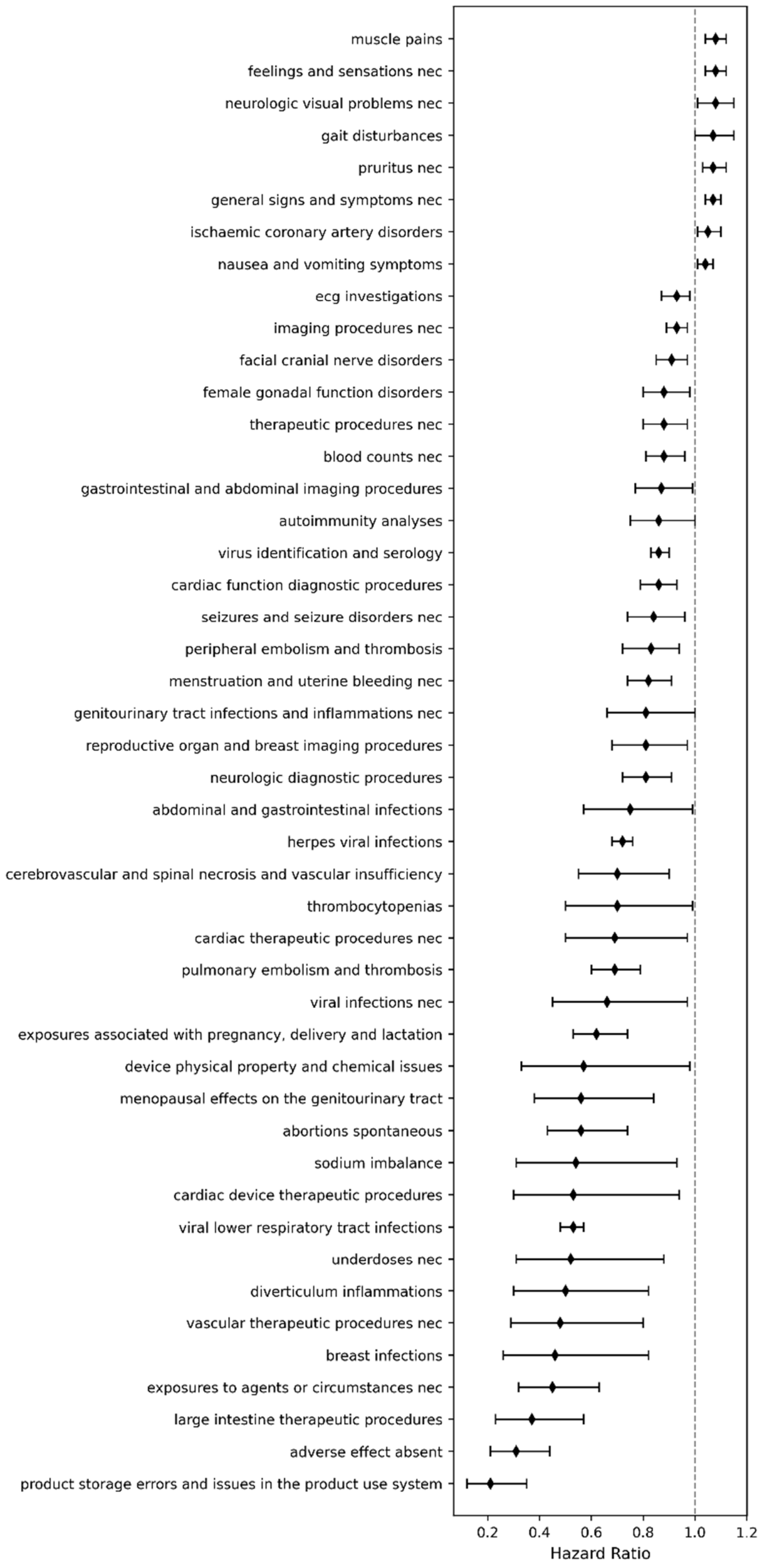

	N	HR	95% CI	P
muscle pains	3822	1.08	(1.04, 1.12)	<.0001
feelings and sensations nec	3245	1.08	(1.04, 1.12)	<.0001
neurologic visual problems nec	917	1.08	(1.01, 1.15)	<.05
gait disturbances	930	1.07	(1.00, 1.15)	<.05
pruritus nec	2898	1.07	(1.03, 1.12)	<.001
general signs and symptoms nec	6709	1.07	(1.04, 1.10)	<.0001
ischaemic coronary artery disorders	2745	1.05	(1.01, 1.10)	<.05
nausea and vomiting symptoms	6933	1.04	(1.01, 1.07)	<.01
ecg investigations	1601	0.93	(0.87, 0.98)	<.05
imaging procedures nec	2665	0.93	(0.89, 0.97)	<.01
facial cranial nerve disorders	974	0.91	(0.85, 0.97)	<.01
female gonadal function disorders	398	0.88	(0.80, 0.98)	<.05
therapeutic procedures nec	438	0.88	(0.80, 0.97)	<.05
blood counts nec	946	0.88	(0.81, 0.96)	<.01
gastrointestinal and abdominal imaging procedures	265	0.87	(0.77, 0.99)	<.05
autoimmunity analyses	218	0.86	(0.75, 1.00)	<.05
virus identification and serology	3281	0.86	(0.83, 0.90)	<.0001
cardiac function diagnostic procedures	658	0.86	(0.79, 0.93)	<.001
seizures and seizure disorders nec	229	0.84	(0.74, 0.96)	<.05
peripheral embolism and thrombosis	246	0.83	(0.72, 0.94)	<.01
menstruation and uterine bleeding nec	391	0.82	(0.74, 0.91)	<.001
genitourinary tract infections and inflammations nec	95	0.81	(0.66, 1.00)	<.05
reproductive organ and breast imaging procedures	130	0.81	(0.68, 0.97)	<.05
neurologic diagnostic procedures	323	0.81	(0.72, 0.91)	<.001
abdominal and gastrointestinal infections	65	0.75	(0.57, 0.99)	<.05
herpes viral infections	1311	0.72	(0.68, 0.76)	<.0001
cerebrovascular and spinal necrosis and vascular insufficiency	64	0.7	(0.55, 0.90)	<.01
thrombocytopenias	35	0.7	(0.50, 0.99)	<.05
cardiac therapeutic procedures nec	38	0.69	(0.50, 0.97)	<.05
pulmonary embolism and thrombosis	247	0.69	(0.60, 0.79)	<.0001
viral infections nec	26	0.66	(0.45, 0.97)	<.05
exposures associated with pregnancy, delivery and lactation	170	0.62	(0.53, 0.74)	<.0001
device physical property and chemical issues	13	0.57	(0.33, 0.98)	<.05
menopausal effects on the genitourinary tract	24	0.56	(0.38, 0.84)	<.01
abortions spontaneous	58	0.56	(0.43, 0.74)	<.0001
sodium imbalance	13	0.54	(0.31, 0.93)	<.05
cardiac device therapeutic procedures	12	0.53	(0.30, 0.94)	<.05
viral lower respiratory tract infections	588	0.53	(0.48, 0.57)	<.0001
underdoses nec	14	0.52	(0.31, 0.88)	<.05
diverticulum inflammations	16	0.5	(0.30, 0.82)	<.01
vascular therapeutic procedures nec	15	0.48	(0.29, 0.80)	<.01
breast infections	12	0.46	(0.26, 0.82)	<.01
exposures to agents or circumstances nec	34	0.45	(0.32, 0.63)	<.0001
large intestine therapeutic procedures	24	0.37	(0.23, 0.57)	<.0001
adverse effect absent	30	0.31	(0.21, 0.44)	<.0001
product storage errors and issues in the product use system	13	0.21	(0.12, 0.35)	<.0001

Figure S3. *Cont.*
